# 3D-printed titanium scaffolds coated with a multifunctional photothermal-responsive hydrogel promote osteoporotic bone defect repair

**DOI:** 10.1016/j.mtbio.2026.102879

**Published:** 2026-01-29

**Authors:** Chenchen Wang, Yuan Wang, Xiaojun Li, Hao Cao, Chenfeng Wang, Sheng Han, Haotian Chen, Xin Zhao, Shude Yang

**Affiliations:** a Department of Chemical Engineering and Energy Technology, Shanghai Institute of Technology, Shanghai, 201418, China; b Center of Plastic and Cosmetic Surgery, School and Hospital of Stomatology, China Medical University, Liaoning Provincial Key Laboratory of Oral Diseases, Shenyang, Liaoning, 110002, China; c Department of Plastic Surgery, The First Hospital of China Medical University, Shenyang, Liaoning, 110011, China; d Shanghai Key Laboratory of Orthopaedic Implants, Department of Orthopaedic Surgery, Shanghai Ninth People's Hospital, Shanghai Jiao Tong University School of Medicine, No. 639 Zhizaoju Road, Shanghai, 200011, China

**Keywords:** Hydrogel coating, Titanium scaffolds, Photothermal-responsion, Cellular senescence, Osteoporotic bone defect

## Abstract

Osteoporotic bone defects are difficult to repair since conventional titanium scaffolds lack bioactivity and cannot overcome impaired osteogenesis, chronic inflammation, oxidative stress, and cellular senescence. To address these limitations, a multifunctional tannic acid (TA)-based hydrogel coating was developed for 3D-printed titanium alloy scaffolds. The hydrogel was formed from TA, acrylamide, and 3-acrylamidophenylboronic acid, with osteogenic growth peptide (OGP) as a bioactive component. Prussian blue (PB) nanoparticles and quercetin (QUE) were incorporated to provide dynamic crosslinking, photothermal conversion, antioxidant, and anti-inflammatory functions. Stromal cell-derived factor-1α (SDF-1α) was further integrated to recruit endogenous stem cells. The coating exhibited enhanced antiwear and self-healing properties, while near-infrared irradiation (NIR) triggered PB-mediated photothermal effects, thereby improving biotribological performance and accelerating self-repair. *In vitro*, the hydrogel coating combined with NIR promoted bone marrow mesenchymal stem cell migration, adhesion, and osteogenic differentiation, while simultaneously scavenging reactive oxygen species, attenuating inflammation, reducing cellular senescence, and inducing M2 macrophage polarization. *In vivo*, the hydrogel coated 3D-printed titanium scaffold markedly enhanced osteoporotic bone defect repair. Overall, this multifunctional hydrogel coating transforms passive titanium scaffolds into bioactive implants, offering a promising strategy for promoting osteoporotic bone regeneration.

## Graphical abstract

**Figure:**
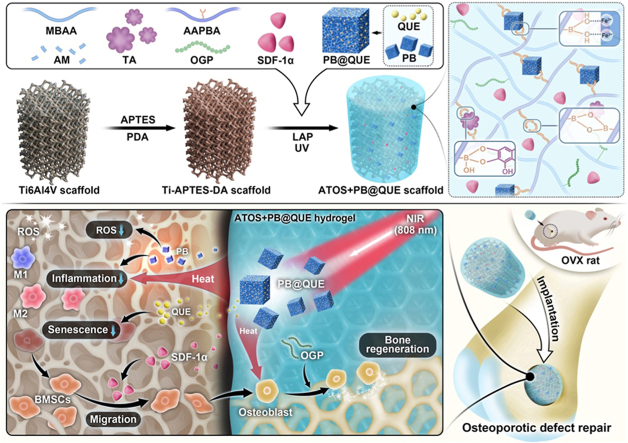

## 1 Introduction

Osteoporosis, a systemic metabolic disease affecting over 200 million people worldwide, significantly increases the risk of fractures, particularly among the elderly [1]. Fracture healing in osteoporotic patients is often impaired due to reduced bone regenerative capacity, resulting in persistent bone defects and increased therapeutic challenges. The management of osteoporotic bone defects remains challenging, largely owing to diminished bone formation potential, abnormal inflammatory responses, and a harsh senescent microenvironment (SME) [2]. These defects disrupt bone structural integrity, compromise mechanical stability, and lead to bone atrophy and deformity, ultimately impairing function and reducing quality of life [3]. Therefore, developing effective therapeutic strategies for osteoporotic bone defects remains an urgent clinical need. Currently, autologous bone grafting remains the gold standard for bone defect repair [4]. However, its clinical application is severely limited by the scarcity of donor tissue, especially in cases involving large-segment bone defects and elderly patients. As alternatives, biomaterial grafts have attracted considerable attention, with titanium-based biomedical materials showing particularly promising prospects [5,6]. Titanium and its alloys are widely used in orthopedic surgery to treat bone defects, such as large-segment bone defects and tumor-resected defects [7,8]. This is mainly attributed to their excellent biocompatibility, long-term stability associated with strong corrosion resistance, and favorable mechanical properties, such as a high strength-to-weight ratio and high fracture toughness [[9], [10], [11]]. Nevertheless, several challenges hinder their clinical performance. Abrasion between titanium scaffolds and host bone generates wear particles, which may induce metallosis, impair bone regeneration, provoke immunoinflammatory responses, promote bone resorption, and even lead to chronic inflammation [12,13]. In addition, the elastic modulus of titanium alloys is significantly higher than that of native bone, resulting in a “stress shielding” effect. This phenomenon disrupts physiological load transfer to the surrounding bone matrix, thereby compromising implant–bone interface stability [14]. Moreover, titanium and its alloys inherently lack osteoinductive properties, leading to insufficient new bone formation within the scaffold and ultimately unsatisfactory repair outcomes [15]. Given these limitations, surface modification of titanium alloys has become essential, with the primary goals of improving their tribological performance, mitigating inflammatory responses, and enhancing osteogenic capacity.

SME represents another prominent feature of osteoporotic bone defects. A hallmark of the SME is the excessive accumulation of reactive oxygen species (ROS), which enhances osteoclast activity while impairing osteoblast function [16]. Mechanistically, ROS have been shown to suppress the Wnt/β-catenin signaling pathway and β-catenin expression while activating FOXO transcription factors, thereby inhibiting the osteogenic differentiation of bone mesenchymal stem cells (BMSCs) [17]. In addition, the SME is characterized by chronic, low-grade inflammation [18]. A central contributor to this state is the senescence-associated secretory phenotype (SASP), a heterogeneous and dynamic secretome produced through interactions between senescent cells and their microenvironment [19,20]. Pro-inflammatory cytokines, including tumor necrosis factor-α (TNF-α), interleukin-1β (IL-1β), IL-6, and IL-17, exacerbate bone loss by promoting osteoclast differentiation and activation [21]. Beyond this, these cytokines further compromise the osteogenic potential of primary BMSCs [22]. In addition, autophagy, a cellular process responsible for maintaining homeostasis through continuous proteome turnover and the removal of dysfunctional proteins, plays a protective role against cellular senescence. Therefore, developing biomaterials capable of modulating the SME is highly desirable, particularly in terms of alleviating oxidative stress, suppressing inflammation, delaying senescence, and promoting autophagy.

Hydrogels have been widely investigated as bioactive materials for bone repair due to their excellent lubricating properties and their ability to provide a biocompatible environment conducive to osteogenesis [[23], [24], [25], [26]]. Their high tunability allows hydrogels to exhibit favorable mechanical and tribological properties while dissipating interfacial stress, thereby maintaining stable connections within bone tissue networks [27]. Tannic acid (TA), a polyphenol-rich natural polysaccharide monomers, possesses diverse pharmacological activities, including anti-inflammatory and antioxidant effects [28], and has been extensively employed as a key component in hydrogel fabrication [29]. However, TA-based hydrogels require further optimization, as their acidic microenvironment is not well-suited for bone regeneration and their relatively low mechanical strength limits long-term in vivo applications. To address these shortcomings, osteogenic growth peptide (OGP), a well-known osteoinductive molecule, can be incorporated into hydrogels to endow them with bioinductive activity and enhance bone regeneration [30]. Through its surface-active groups, OGP can chemically interact with functional groups within the hydrogel network, thereby providing a favorable microenvironment for early BMSC adhesion and osteogenic differentiation [31]. Moreover, the incorporation of three-dimensional (3D) nanomaterials, such as Prussian blue (PB), has proven effective in reinforcing the mechanical and tribological properties of hydrogels [32]. PB stands out as a photothermal nanomaterial with exceptional antioxidant activity and efficient photothermal conversion under near-infrared irradiation (NIR) [[33], [34], [35]]. The photothermal effect of PB enhances molecular motion within the hydrogel network, thereby facilitating the self-healing of structural damage [36]. In addition, PB nanozymes are biocompatible and possess intrinsic ROS-scavenging activity, while their hollow internal architecture procides a natural platform for therapeutic drug loading [37].

Additionally, further modification of hydrogels is required to more effectively eliminate senescent cells, regulate the SME, enhance autophagy, and promote stem cell migration and osteogenic differentiation. Quercetin (QUE), a flavonoid compound with well-documented anti-inflammatory and antioxidant activities [38], has shown great potential in treating aging-related diseases [39]. Importantly, QUE can selectively eliminate senescent cells in vivo and exert anti-inflammatory effects during the early stages of bone implantation [40]. To achieve controlled and sustained release, QUE was loaded into the hollow interior of PB nanoparticles via a co-precipitation method to generate PB@QUE nanoparticles, which were subsequently encapsulated into the hydrogel network. Stromal cell-derived factor-1 (SDF-1), the only known ligand of the chemokine receptor C-X-C chemokine receptor type 4 (CXCR4), plays a central role in cell adhesion [41]. SDF-1α, in particular, has been reported to promote autophagy, induce BMSC chemotaxis, and enhance osteogenic differentiation via the Wnt/β-catenin signaling pathway, making it an ideal osteogenic regulator [42]. Therefore, SDF-1α was directly encapsulated into the hydrogel to enable localized and sustained release at the defect site, facilitated by the inherent swelling properties of hydrogels. In summary, a base hydrogel network was fabricated through the copolymerization of TA and 3-acrylamidophenylboronic acid (AAPBA) with acrylamide (AM) and ostegenic growth peptide (OGP) via dynamic boronate ester bonds. The hydrogel network was constructed with covalent boronate ester bonds and dense hydrogen bonds as non-degradable crosslinking units, enabling long-term lubricity and durability at the bone repair interface [43,44]. SDF-1α was incorporated to modulate cell adhesion, while PB nanoparticles served as additional crosslinking sites, drug carriers, and photothermal agents. The composite sol was then filled into titanium scaffolds via a sol-gel method to form the final coating.

**Scheme 1:**
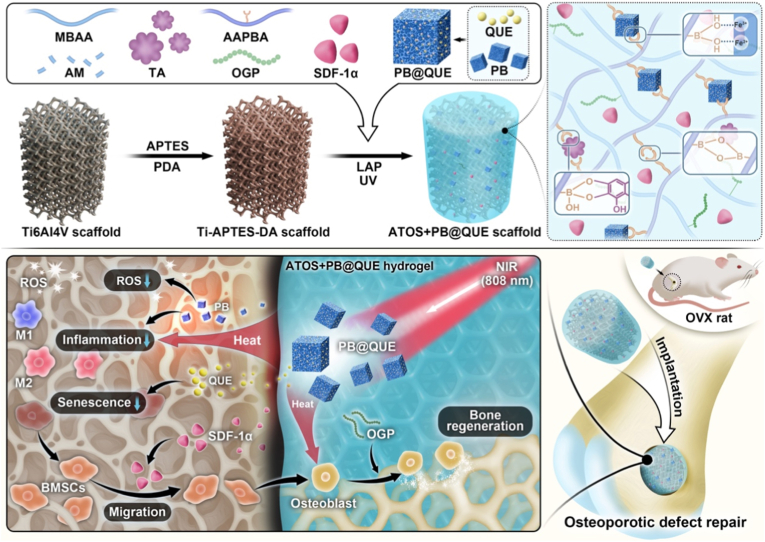
A photothermal-responsive, bioactive hydrogel coating with an integrated drug delivery system was applied to titanium alloy scaffolds. This multifunctional coating enables controlled drug release at the defect site, which suppresses inflammation, scavenges ROS, delays cellular senescence, and prompts BMSCs osteogenic differentiation. These effects accelerate the repair of osteoporotic bone defects in rats.

In this study, we developed a photothermal-responsive, wear-resistant TA-based hydrogel coating integrated with a drug delivery system on a 3D-printed titanium alloy scaffold to address the multifactorial barriers of osteoporotic bone defect repair. The coating integrates PB nanoparticles and QUE for photothermal, antioxidant, and anti-inflammatory regulation, and incorporates SDF-1α to promote stem cell recruitment and osteogenesis. We hypothesize that this photothermal-responsive, self-healing coating not only improves the tribological performance of titanium alloy scaffolds but also suppresses inflammation, scavenges ROS, delays cellular senescence, and promotes BMSC osteogenic differentiation, thereby creating a favorable osteogenic microenvironment for bone regeneration (Scheme 1). This strategy transforms conventional titanium scaffolds into bioactive implants, offering a promising approach for osteoporotic bone defect regeneration.

## 2 Results and discussion

### 2.1 Evaluation of PB@QUE nanoparticles

**Fig. 1:**
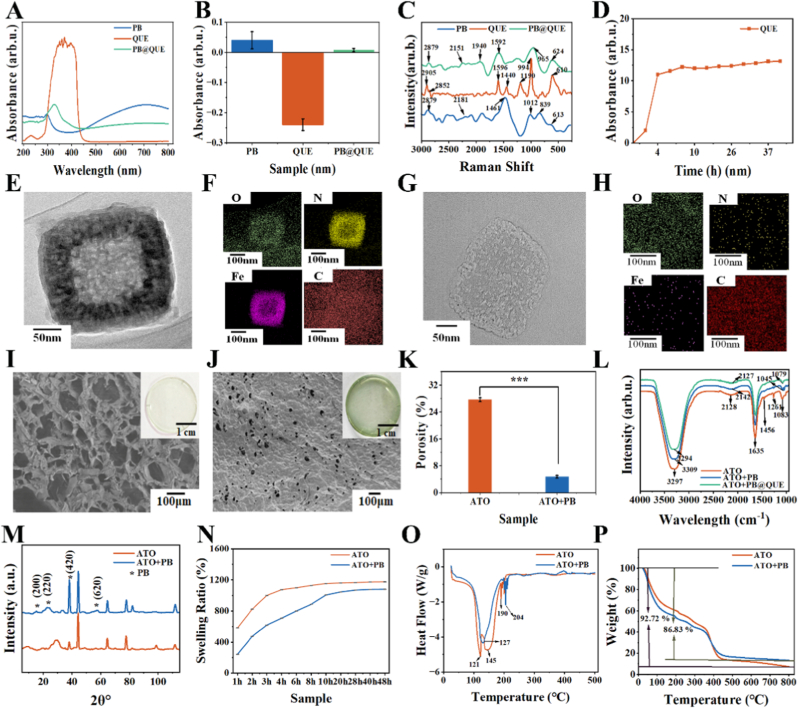
Structural and morphological characterization of PB@QUE nanoparticles and different hydrogel coatings. (A) UV-visible absorption spectra, (B) Zeta potential profiles and (C) Raman spectra of PB@QUE. (D) Release kinetics of QUE from PB@QUE in PBS (pH = 7.4). (E) TEM images of hollow PB nanoparticles and (F) corresponding elemental mapping. (G) TEM images of PB@QUE nanoparticles and (H) corresponding elemental spectra. (I–J) SEM images of ATO (I) and ATO + PB (J) hydrogel coating, and inset images show the macroscopic appearance of the samples. (K) Porosity analysis, (L) infrared spectroscopy, (M) Raman shift, (N) swelling ratios, (O) differential scanning calorimetry (DSC), and (P) thermogravimetric analysis (TGA) of the hydrogel coatings.

According to previous reports, PB at a concentration of 40 μg/mL is not only non-toxic but can also achieve an effective photothermal response, with the temperature rise to 40 °C under NIR [40,45]. Based on this, QUE-loaded PB nanoparticles (PB@QUE) were prepared by a co-precipitation method in this study. The encapsulation efficiency (EE) was calculated to be 75 % by measuring the absorbance at 378 nm before and after QUE loading via ultraviolet spectroscopy (Fig. S1A and S1B). The characteristic peak of QUE at 378 nm disappeared in PB@QUE in the ultraviolet (UV)-visible absorption spectra (Fig. 1A), indicating that QUE was successfully loaded onto PB through interactions. Meanwhile, the characteristic peak of PB at 300 cm^−1^ exhibited a red shift to 332 cm^−1^ in PB@QUE, accompanied by increased absorbance, confirming the formation of a stable composite structure without disrupting the basic chemical structures of PB and QUE. Zeta potential measurements revealed that PB was positively charged, QUE was negatively charged, and PB@QUE exhibited a weakly positive potential (Fig. 1B). This charge neutralization phenomenon further verified the successful loading of QUE onto PB without damaging their intrinsic structures. In Raman spectroscopy, the characteristic peak of C ≡ N stretching vibration of PB at 2181 cm^−1^ and the Fe–N/Fe–C bending vibration peak at 613 cm^−1^ both shifted to 2151 cm^−1^ and broadened to 624 cm^−1^ in the spectrum of PB@QUE (Fig. 1C). In addition, the characteristic peaks of QUE were retained in the spectrum of PB@QUE. For example, the benzene ring skeleton vibration peak at 1596 cm^−1^ and the peak of ortho-disubstitution of the benzene ring at 994 cm^−1^ were retained, whereas the C–O stretching vibration peaks at 1190 and 1400 cm^−1^ were weakened and broadened. The cumulative release rate of QUE reached a stable level of approximately 12 % at around 10 h (Fig. 1D). In addition, electron microscopy images clearly show that the hollow structure of PB was filled with QUE, while the particle size remained unchanged before and after loading (Fig. 1E–H). The Fe elemental framework of PB remained unchanged after loading, and N and O elements were distributed within the PB structure (Fig. 1F and H). The infrared data (Fig. S1C), together with the above results, further confirmed that QUE was successfully loaded into the hollow structure of PB. These results indicated that PB and QUE formed a composite structure through surface interactions. While retaining the basic framework of PB and the molecular structure of QUE, the effective loading of QUE was achieved, thus ensuring the synergistic effect of the photothermal performance of PB and the pharmacological activity of QUE.

### 2.2 Characterization of PB@QUE-Incorporated hydrogel coatings

Upon the introduction of PB, the color of the AM/AAPBA/TA/MBAA/OGP (ATO) hydrogel coating changed from yellow to blue (Fig. 1I and J). Correspondingly, compared with the ATO hydrogel coating (D = 57.01, Fig. S2A), the ATO + PB hydrogel coating (D = 10.92, Fig. S2B) showed a significant decrease in both pore size and pore number, with the porosity reduced by 82.97 % (Fig. 1K). Fourier transform infrared (FTIR) spectroscopy was used to confirm the chemical structures of ATO, ATO + PB, and ATO + PB@QUE hydrogel coatings (Fig. 1L). A characteristic absorption peak at 1635 cm^−1^ was observed, corresponding to the –CONH– group generated by the reaction between AM monomers and OGP. Meanwhile, the stretching vibration of B–O–C bonds in the boronate ester linkage formed between TA and AAPBA was observed at 1456 cm^−1^. The coexistence of these characteristic peaks clearly indicated the successful polymerization of the ATO base network. Furthermore, Fe–CN groups of PB nanoparticles interacted with hydroxyl and boronic acid components (TA and AAPBA) in the hydrogel coating. These interactions disrupted C–O vibrational environment, causing the peak at 1261 cm^−1^ to disappear and the peak at 1083 cm^−1^ to split in ATO + PB. In the ATO + PB@QUE coating, a broad peak was observed at 1079 cm^−1^. This was attributed to the phenolic hydroxyl groups of QUE forming multiple hydrogen bonds with the hydrogel network (TA and AAPBA) and the surface of PB, thereby reconstructing the chemical environment around C-O bonds and homogenizing the distribution of vibrational modes. The swelling ratios of both hydrogel coatings reached equilibrium after 20 h. Specifically, the swelling ratio of the ATO + PB coating was 9.87 % lower than that of the ATO coating, confirming that PB increased the cross-linking density and restricted the water absorption capacity of the hydrogel matrix. These results further confirmed that PB, acting as a cross-linking point, enhanced the cross-linking density of the hydrogel coating. The X-ray diffraction (XRD) patterns of ATO and ATO + PB hydrogel coatings revealed clear differences in crystal structures between the two samples (Fig. 1M). In the ATO + PB hydrogel coating, typical diffraction peaks corresponding to PB crystals were abserved, with specific assignments as follows: 2θ ≈ 15.3° corresponding to the (200) crystal plane, 23.6° corresponding to the (220) crystal plane, 2θ ≈ 38.1° corresponding to the (420) crystal plane, and 57.5° corresponding to the (620) crystal plane [46,47]. The presence of these characteristic peaks directly confirmed that PB nanoparticles were successfully introduced into the hydrogel system while maintaining their intrinsic crystal structure within the network. Among them, the diffraction peak corresponding to the (420) crystal plane (2θ ≈ 38.1°) already existed in the original ATO hydrogel coating, which was attributed to the local ordering of stacked TA polyphenol rings. Upon the introduction of PB, this peak exhibited a “synergistic diffraction” effect with PB crystals, leading to a significant enhancement in peak intensity. Both ATO and ATO + PB reached swelling equilibrium after 24 h; the swelling ratio of ATO + PB was 1068 %, which was 8.2 % lower than that of ATO (Fig. 1N). This result indicated that ATO + PB could still achieve effective drug release through swelling. In the differential scanning calorimetry (DSC) curves, the reduced number of exothermic peaks and their shifted positions indicated that PB, acting as a cross-linking site, formed coordination bonds and strong hydrogen bonds with the phenolic hydroxyl groups of TA and boronic acid groups of AAPBA in the hydrogel network, thereby replacing the original interaction network dominated by weak hydrogen bonds (Fig. 1O). The crystalline framework of PB nanoparticles served as a “rigid support,” restricting the thermal motion of hydrogel polymer chains and significantly increasing the thermal decomposition energy barrier of the hydrogel network. This shift moved the main endothermic peak of ATO + PB from 190 °C to 204 °C, indicating a marked enhancement in thermal stability. The thermogravimetric analysis (TGA) further demonstrated the strengthening effect of PB on the thermal stability of ATO hydrogel coatings (Fig. 1P). In the 0–400 °C range, ATO + PB hydrogel coatings exhibited a faster weight loss rate than ATO. This behavior was attributed to the thermal cleavage of weak interactions generated by PB at medium and low temperatures, which accelerated the decomposition of the hydrogel network and caused faster weight loss. A contrasting trend emerged above 400 °C. ATO showed a significantly faster weight loss rate, while ATO + PB ultimately achieved a higher residual mass (92.72 %) compared with ATO (86.83 %). This discrepancy can be explained by high-temperature decomposition behavior. In ATO, although the carbonization of polyphenols and polymers formed partial residues, these carbonized products remained prone to further decomposition at high temperatures. However, in ATO + PB, the interactions between PB and organic components remained more stable at high temperatures and were more resistant to thermal cleavage. Moreover, the intrinsic high-temperature resistance of the Fe-CN framework further reduced mass loss caused by decomposition in PB. Under the combined effects of these two factors, ATO + PB ultimately attained a higher residual mass.

### 2.3 Mechanical and photothermal properties of hydrogel coatings

**Fig. 2:**
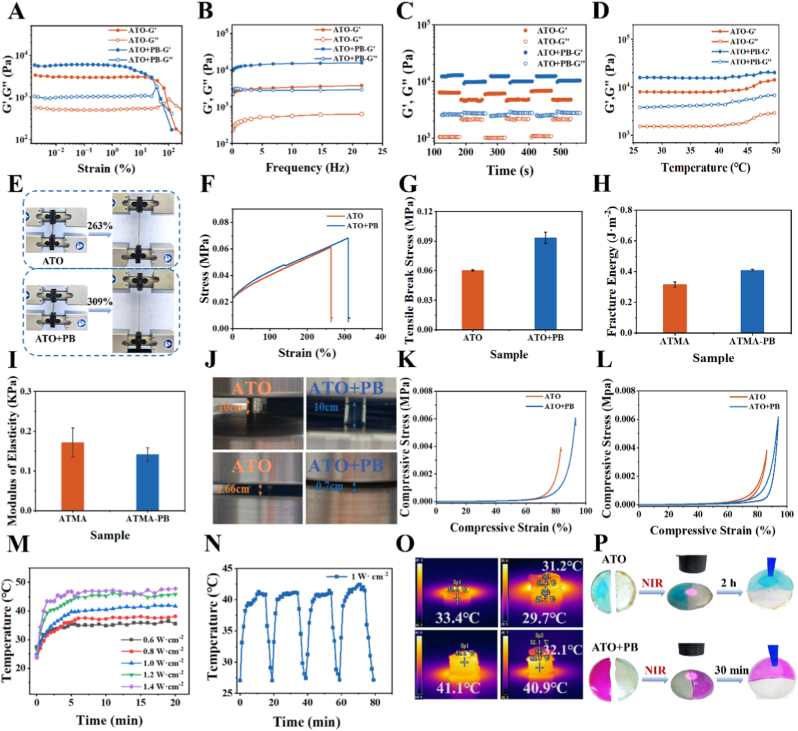
Mechanical and photothermal properties of different hydrogel coatings. (A) Strain sweep tests to determine the linear viscoelastic region. (B) Frequency sweep tests. (C) Time sweep tests. (D) Temperature sweep tests. (E) Photographs showing tensile deformation. (F) Tensile stress-strain curves. (G) Maximum tensile fracture stress. (H) Maximum fracture energy. (I) Tensile Young's modulus. (J) Photographs showing compressive deformation. (K) Compressive stress-strain curves. (L) Three-cycle compressive stress-strain curves. (M) Photothermal heating curves under near-infrared irradiation (NIR) at different power densities. (N) Cyclic photothermal heating of ATO + PB under NIR at 1 W cm^−2^. (O) Infrared thermal images (top left: 3D-printed object; top right: 3D-printed object covered with pigskin; bottom left: ATO + PB hydrogel coating; bottom right: ATO + PB hydrogel coating covered with pigskin). (P) Photographs comparing the self-healing behavior of ATO and ATO + PB hydrogel coatings under NIR stimulation.

Through tensile, compression, and rheological tests, significant differences in mechanical properties between ATO and ATO + PB hydrogel coatings were observed. These differences were primarily attributed to the dual roles of PB as both a crosslinker and a rigid support within the ATO hydrogel network. In dynamic rheological measurements, the storage modulus (G′) of ATO + PB was remarkably higher than that of ATO (Fig. 2A). Moreover, the yield point in the G′-strain curve shifted to higher strain values, and the modulus decline at high strains became more gradual. These results indicated that PB incorporation enhanced the shear resistance of the hydrogel network, in which boronate bonds and coordination interactions contributed to improve elastic recovery. The frequency sweep tests, both hydrogels showed a slight increase in G′ and loss modulus (G″) with increasing frequency (Fig. 2B). Notably, the G′ value of ATO + PB (13,280 Pa) was 4.3 times higher than that of ATO (3019 Pa), confirming that the PB-induced crosslinked networks provided more stable elasticity and pronounced elastic solid-like behavior. The self-healing capability of the hydrogels was further evaluated using dynamic recovery tests at room temperature (Fig. 2C). Owing to the dynamic boronate ester bonds between TA catechol groups and phenylboronic acid moieties, ATO inherently exhibited self-healing behavior. For ATO + PB, both G′ and G″ values became higher and more stable compared with those of ATO, demonstrating that PB further enhanced the self-healing performance of the hydrogel coating. To investigate the influence of the near-infrared photothermal properties of PB on the mechanical stability of the hydrogel coatings, temperature sweep tests were conducted (ɣ = 1 %, 25–50 °C, Fig. 2D). When the temperature exceeded 45 °C, G′ and G″ of both hydrogels increased slowly, suggesting that the photothermal temperature should be maintained below 45 °C to preserve mechanical integrity. Throughout the temperature range, the ATO + PB hydrogel consistently maintained higher G′ and G″ values than ATO, indicating that the strong coordination and hydrogen bonding interactions, together with the rigid framework provided by PB, effectively enhanced the thermal softening resistance of the hydrogel network. Collectively, PB incorporation synergistically enhanced multiple mechanical properties of the hydrogel coatings, including anti-deformation capability, elastic stability, self-healing efficiency, and thermal resistance, thereby supporting their suitability for in vivo and photothermally responsive applications.

The tensile properties of the hydrogel coatings were further evaluated (Fig. 2E and F). Upon PB incorporation, the tensile strain increased from 263 % for ATO to 309 % for ATO + PB. Correspondingly, the maximum tensile stress increased from 0.06 MPa to 0.09 MPa (Fig. 2G), while the fracture energy rose from 0.315 J m^−2^ to 0.41 J m^−2^ (Fig. 2H). In contrast, the Young's modulus of ATO + PB slightly decreased from 0.17 KPa to 0.14 Kpa compared with ATO (Fig. 2I). Overall, PB incorporation improved both the tensile strength and toughness of the hydrogel coating while moderately reducing stiffness, rendering the material more adaptable to the dynamic mechanical environment of living tissues. Representative photographs illustrating compressive strain were shown in Fig. 2J. Under the same conditions, the maximum compressive strain of ATO reached 83.5 %, whereas ATO + PB reached a higher compressive strain of 93.1 % (Fig. 2K). Cyclic compression tests further demonstrated that ATO could be repeatedly compressed three times at 83 % strain, while ATO + PB maintained structural integrity over three cycles at 93 % strain (Fig. 2L). These results indicated that the PB-reinforced crosslinked network significantly improved compressive deformation ability, cyclic stability, and compressive strength of the hydrogel coatings. Through the above tests, it was proved that the mechanical properties and thermal stability of ATO + PB were enhanced, and it could meet the basic in vitro tests.

The photothermal response of the hydrogel coatings was then evaluated under 808 nm NIR light. The temperature profiles of ATO + PB under different power densities are shown in Fig. 2M. A power density of 1 W cm^−2^ was selected as optimal, as it increased the temperature of the ATO + PB hydrogel coating to approximately 41 °C, which is suitable for biomedical applications. Under this irradiation condition, repeated heating–cooling cycle were performed 4 times (Fig. 2N). The ATO + PB hydrogel coating rapidly reached 41 °C within 10 min and exhibited stable photothermal behavior, while cooling back to room temperature within 5 min after the cessation of irradiation, with temperature fluctuation below 5 °C, demonstrating the excellent photothermal stability of the ATO + PB hydrogel coating.

Considering the requirement for light penetration during in vivo applications, thermal imaging was performed to monitor the temperature changes of ATO + PB under NIR light through porcine skin (Fig. S3). The presence of porcine skin had minimal influence on NIR penetration (Fig. 2O), and ATO + PB could still reach the target temperature after 15 min (Fig. S3H and S3P). To evaluate self-healing behavior, both ATO and ATO + PB hydrogel coatings were cut into two halves, with one half stained to facilitate visualization of the healing interface. Owing to the presence of dynamic boronate bonds, ATO exhibited self-healing behavior at room temperature, requiring approximately 3 h for complete recovery (Fig. S4A). In contrast, the healing time of ATO + PB was significantly shortened to approximately 1 h (Fig. S4B). This improvement was attributed to the additional crosslinking provided by PB, which helped preserve network integrity at the damaged interface and facilitated the reformation of boronate ester bonds by reducing excessive molecular disorder. Self-healing behavior under NIR irradiation was further investigated. Continuous irradiation of the fracture site at 1 W cm^−2^ reduced the healing time of ATO by approximately 1 h (Fig. 2P). In contrast, the healing time of ATO + PB was further reduced to 30 min. The enhanced thermal conductivity and photothermal conversion efficiency of PB promoted more uniform energy distribution within the hydrogel network, while the additional crosslinking points facilitated molecular chain diffusion and rearrangement, thereby substantially accelerating the self-healing process.

### 2.4 Tribological properties of hydrogel coatings in vitro

**Fig. 3:**
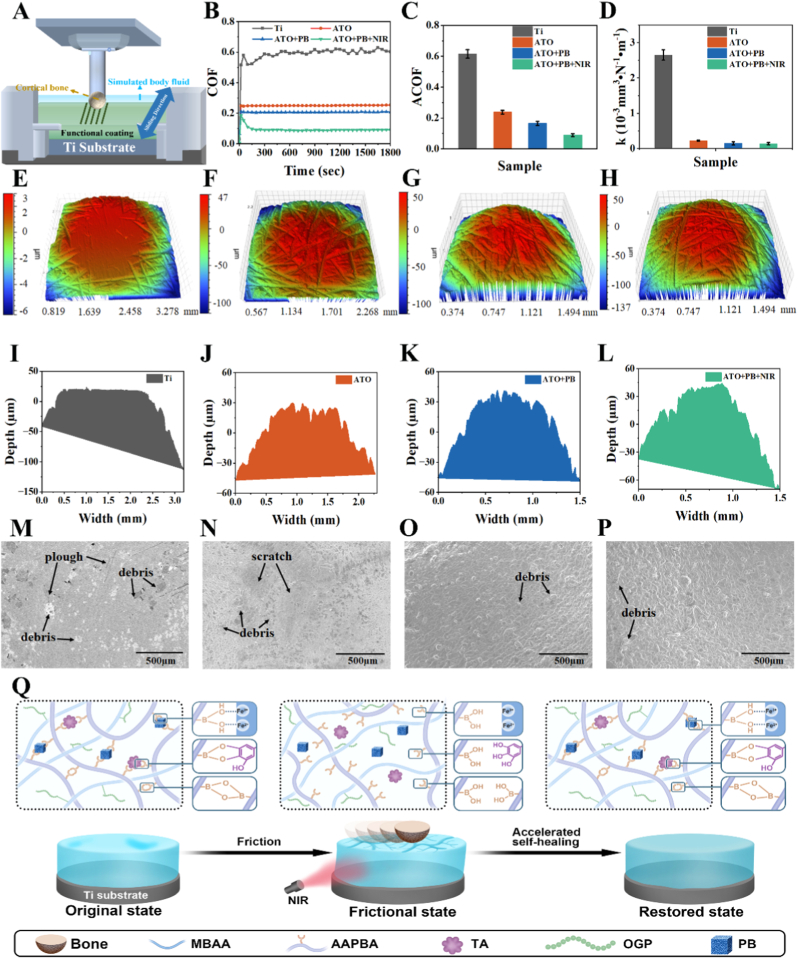
Tribological performance of different hydrogel coatings. (A) Schematic illustration of the friction test setup. (B) Friction coefficient curves. (C) Average friction coefficients. (D) Wear rates. (E–H) Three-dimensional white-light interferometry images and (I–L) corresponding wear depths of the upper friction counterparts (bone balls) after wear for all samples. (E, I) Ti plate; (F, J) ATO coating; (G, K) ATO + PB coating; (H, L) ATO + PB + NIR coating. (M − P) SEM images of the hydrogel coatings after wear at 100× magnification, (M) Ti plate; (N) ATO coating; (O) ATO + PB coating; (P) ATO + PB + NIR coating. (Q) Schematic illustration of the tribological mechanisms of hydrogel coatings.

Given that bone defect implant scaffolds are subjected to long-term dynamic frictional environments in vivo, the frictional behavior of hydrogel-coated scaffolds was evaluated in simulated body fluid (SBF). Before tribological tests, the interfaces between the Ti6Al4V substrate and the two hydrogel coatings (ATO and ATO + PB) were evaluated. Coating thicknesses were quantified using 3D white light interferometry, yielding average thicknesses of 14.43 μm for the ATO coating and 18.84 μm for the ATO + PB coating (Fig. S5A and S5B). In addition, based on variations in acoustic emission (AE) signals and friction forces, the bonding strengths of the ATO and ATO + PB coatings were quantified as 20.0 N and 20.7 N, respectively (Fig. S5C and S5D). These results indicated that the coating-substrate interfaces exhibited sufficient mechanical stability to withstand the mechanical demands of the implantation procedure [48]. Tribological tests were subsequently performed using a ball-on-plate reciprocating cycle mode to assess the friction and wear performance of the system (Fig. 3A). The friction coefficient (COF) curve of Ti was the highest among all groups, with a maximum average COF (ACOF) of 0.615 (Fig. 3B and C). In contrast, the ATO hydrogel coating exhibited a pronounced lubricating effect, resulting in a significant reduction in both the COF curve and the ACOF (0.238). Following the incorporation of PB, the ACOF was further decreased to 0.165. Notably, the ATO + PB hydrogel coating under NIR exhibited the lowest friction, with an ACOF of 0.089. Compared with Ti, the ACOF of the ATO + PB + NIR group decreased by 85.5 %. Abundant hydrogen bonds were present within the molecular chains of the ATO hydrogel coating, which were proposed to form a hydration layer, thereby reducing the friction coefficient. The rigid structure of PB nanozymes was cross-linked with polymer chains, leading to enhanced shear resistance and improved stress dispersion within the network, which contributed to the friction-reducing effect [49]. Under NIR, photothermal conversion of PB was activated, and the breaking-recombination rate of dynamic boronate bonds was accelerated, facilitating the formation of a more ordered lubricating layer at the friction interface and further lowering the friction coefficient [50].

Both ATO and ATO + PB hydrogel coatings effectively reduced the wear rate (Fig. 3D). Among all groups, ATO + PB + NIR showed the optimal wear performance, with a wear rate of 0.138 × 10^−3^ mm^3^ N^−1^ m^−1^, representing a 94.79 % reduction compared with Ti. 3D white light interferometry images of the upper friction pairs (bone balls) after tribological testing are shown in Fig. 3E–H, with the corresponding wear depth maps presented in Fig. 3I–L. The bone ball paired with Ti exhibited the most severe wear, characterized by the largest wear depth, as well as numerous cracks and debris (Fig. S6A1 and S6A2). Correspondingly, extensive plowing grooves, scratches, and bone debris were observed on the Ti plate surface (Fig. 3M). For the ATO-coated group, wear on both the bone ball and the hydrogel coating was reduced, accompanied by a significant decrease in cracks and debris on the bone ball surface (Fig. 3N, Fig. S6B1 and S6B2). The introduction of PB further enhanced the shear resistance of the coating and enabled a more uniform distribution of frictional stress at the contact interface, resulting in the disappearance of obvious scratches on the ATO + PB hydrogel coating (Fig. S6C1 and S6C2). Among the four coating samples, the ATO + PB + NIR group exhibited the shallowest wear depth and the least amount of wear debris, as evidenced by both 3D white light interferometry and SEM observations (Fig. 3O–P, Fig. S6D1 and S6D2, Fig. S7). Meanwhile, intense energy dissipation caused by local stress concentration was avoided, leading to only minor cracks and limited debris on the bone ball and a substantial alleviation of wear. Adhesive wear during the friction process was further indicated by energy-dispersive spectrometer (EDS) analysis of the corresponding plate surfaces (Fig. S8). This behavior was attributed to the relatively slow self-healing of boronate bonds in the ATO hydrogel at room temperature, where micro-damage could not be rapidly recovered through reversible bond recombination, leading to damage accumulation. After the incorporation of PB, the crosslinking density and mechanical properties of the hydrogel network were enhanced, network integrity was better maintained, and the healing efficiency of boronate bonds was improved, thereby significantly reducing coating wear. Under NIR, the photothermal effect of PB induced a temporary reduction in the spacing of the local polymer network within the hydrogel, promoting stress dispersion during friction and reducing adhesive wear caused by large deformation. In addition, in-situ damage arrest was achieved via photothermal acceleration of self-healing, resulting in only a small amount of debris on the ATO + PB + NIR hydrogel coating [51]. Overall, the ATO + PB hydrogel coating exhibited excellent lubricity and wear resistance, which were enabled by the rigid structure of PB and NIR-induced photothermal conversion. These properties provided a reliable basis for the long-term stability of in vivo implantation. When the bone ball rubbed against the coating, boronate ester bonds between AAPBA and TA or QUE within the coating break were disrupted, resulting in localized structural damage (Fig. 3Q). At room temperature, the healing of these broken bonds proceeded slowly. Under NIR stimulation, the recombination rate of the disrupted bonds was accelerated, thereby promoting coating self-repair. Additionally, NIR facilitated the release of QUE from PB nanoparticles, enabling simultaneous lubrication enhancement and drug delivery.

### 2.5 Biocompatibility and cell adhesion results

**Fig. 4:**
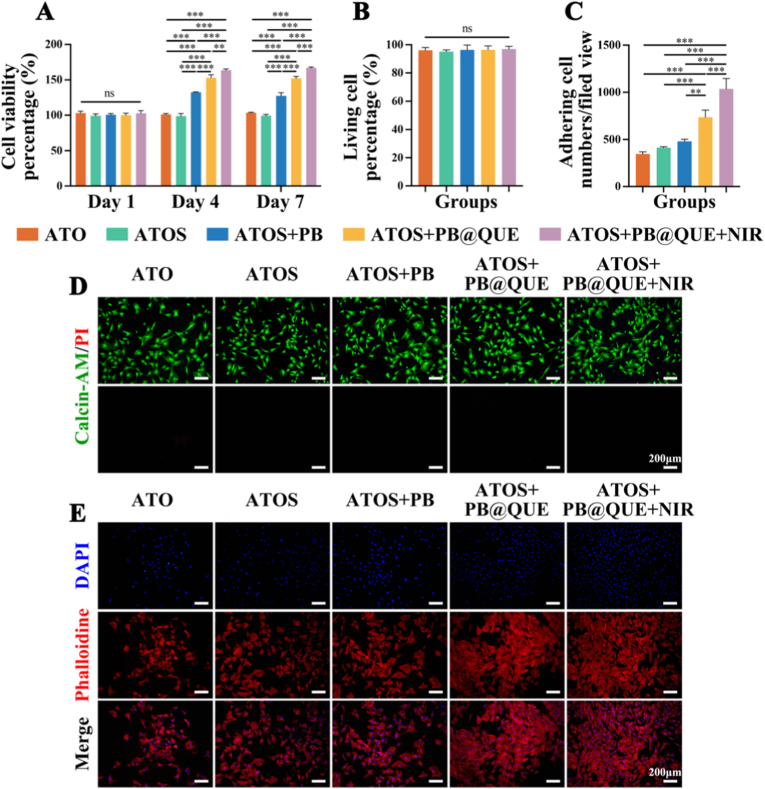
Biocompatibility and cell adhesion of the bone marrow mesenchymal stem cells (BMSCs) in different groups. (A) Cell viability at day 1, 4 and 7 via the CCK-8 assay. (B) Percentage of live cells at day 1 assessed by Live/Dead staining. (C) Proportion of adherent BMSCs. (D) Representative fluorescence images of BMSCs obtained by Live/Dead staining. (E) Representative fluorescence images of adherent BMSCs stained with phalloidin. ∗∗ *p* < 0.01, and ∗∗∗ *p* < 0.001, n = 3.

To assess the biocompatibility of the hydrogel coatings, a CCK-8 assay was first performed. The results of the CCK-8 found that the viability of BMSCs in all the groups increased progressively over the 7-day culture period (Fig. 4A). No significant differences in cell viability were observed among the groups on day 1. In contrast, at days 4 and 7, the cell viabilities of BMSCs cultured with AM/AAPBA/TA/MBAA/OGP/SDF-1α+PB (ATOS + PB), ATOS + PB@QUE, and ATOS + PB@QUE + NIR were obviously higher than those of the other groups. Notably, a gradual increase in cell viability of BMSCs was observed across the ATOS + PB, ATOS + PB@QUE, and ATOS + PB@QUE + NIR groups, in that order. Also, Live/Dead staining was conducted after 1 day of co-culture. Quantitative analysis demonstrated that the ratio of live cells did not differ significantly among the groups (Fig. 4B), which was consistent with the representative fluorescence images (Fig. 4D). Furthermore, extract-based cytotoxicity assays revealed that none of the hydrogel extracts exhibited obvious cytotoxic effects on days 1, 4, or 7 (Fig. S9A). And, extracts from ATOS + PB, ATOS + PB@QUE, and ATOS + PB@QUE + NIR effectively promoted cell proliferation, with ATOS + PB@QUE + NIR showing the most pronounced effect. Consistent with these findings, Live/dead staining results from the extract experiments confirmed high cell viability across all groups (Fig. S9B and S9C), further validating the excellent biocompatibility of the hydrogel coatings. Collectively, these results suggested that the developed hydrogel coatings possess favorable biocompatibility toward BMSCs. SDF-1α is widely distributed in human tissues and organs and is produced and secreted by various cells, including osteoblasts, fibroblasts, and endothelial cells [52]. Accordingly, SDF-1α is considered a biosafe chemokine for BMSCs recruitment. QUE, a naturally bioactive flavonoid, has been investigated in a variety of biomedical applications [53]. In addition, PB nanoparticles with good biosafety have been widely studied in osteogenesis-related biomaterials [54]. Notably, mild heat has been reported to enhance cell proliferation [55]. Taken together, all components incorporated into the hydrogel coatings exhibit excellent biocompatibility.

Cell adhesion is another critical aspect in biomaterial evaluation, as it is a prerequisite for cell differentiation. Accordingly, the adhesion behavior of BMSCs on the hydrogel coatings was evaluated using phalloidin staining. BMSCs cultured on ATOS + PB@QUE and ATOS + PB@QUE + NIR hydrogel coatings exhibited enhanced adhesion and more well-spread cytoskeletal morphology (Fig. 4C and E). Cell adhesion plays an important role in stem cell development to promote bone formation. For instance, integrin receptor alpha 5 beta 1 (α5β1)-mediated cell adhesion is required for the osteoblastic differentiation of BMSCs, thereby promoting bone formation and improving bone repair [56]. Therefore, the ability of the hydrogel coatings to promote BMSC adhesion is expected to contribute to the initiation of osteoblastic differentiation.

### 2.6 BMSCs cell senescence results

**Fig. 5:**
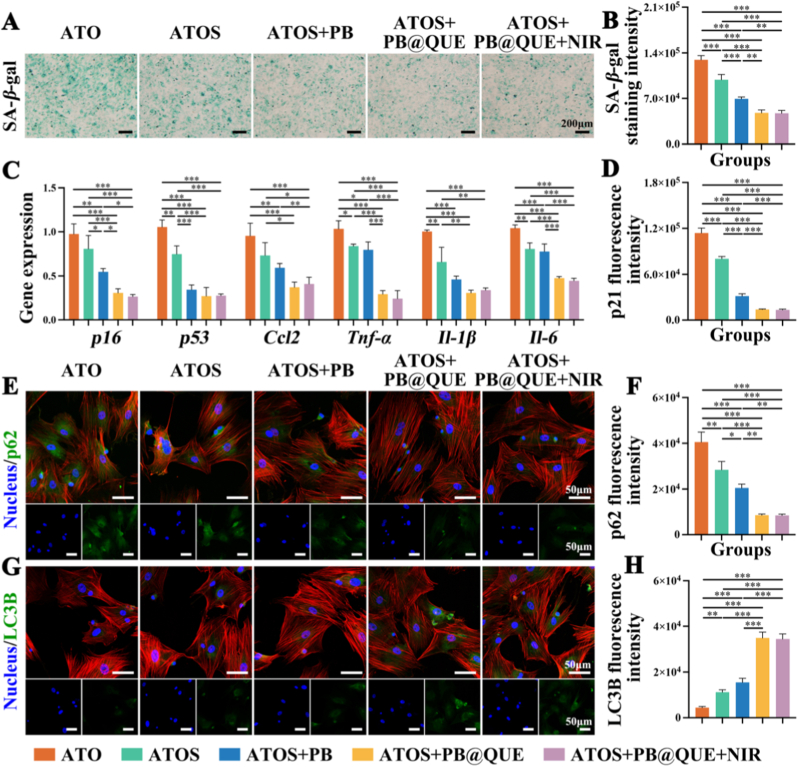
Effects of hydrogel coatings on delaying senescence of BMSCs (A) Representative image of SA-*β*-gal staining. (B) Quantitative analysis of SA-*β*-gal staining intensity. (C) mRNA expression levels of *p16*, *p53*, *Ccl2*, *Tnf-α*, *IL-1β, and IL-6* in BMSCs. (D) Quantitative analysis of p21 fluorescence intensity. (E) Representative fluorescence images of p62 staining. (F) Quantitative analysis of p62 fluorescence intensity. (G) Representative fluorescence images of LC3B staining. (H) Quantitative analysis of LC3B fluorescence intensity. ∗ *p* < 0.05, ∗∗ *p* < 0.01, and ∗∗∗ *p* < 0.001, n = 3.

Next, we explored BMSC senescence following treatment with the different groups. Senescence-associated β-galactosidase (SA-*β*-gal) is a widely recognized marker of cellular senescence [57]. In this study, senescent BMSCs were successfully induced by H_2_O_2_ treatment (Fig. S10). The results of staining analysis indicated that the SA-*β*-gal intensity in the ATOS + PB group was lower than that in the ATO and ATOS groups, suggesting an anti-senescence effect of SDF-1α and PB (Fig. 5A and B). On this basis, the ATOS + PB@QUE and ATOS + PB@QUE + NIR groups exhibited gradually stronger anti-senescence effects compared with the ATOS and ATOS + PB groups, as reflected by the gradually reduced SA-*β*-gal staining intensity. At the transcriptional level, the expression trends of senescence-related genes, including p16 and p53, were highly consistent with the SA-*β*-gal staining results (Fig. 5C). Another senescence-related protein, p21, was further evaluated by immunofluorescence staining. The p21 expression level was markedly reduced in the ATOS + PB@QUE and ATOS + PB@QUE + NIR groups (Fig. S11 and Fig. 5D). Then, senescence-associated secretory phenotype (SASP) expression in BMSCs following treatment with the different groups was examined. SASP refers to a dynamic and diverse set of secreted factors generated through interactions between senescent cells and their surrounding microenvironment. The expression levels of SASP, including *Ccl2*, *Tnf-α*, *Il-1β*, and *Il-6,* were significantly inhibited in the ATOS + PB@QUE and ATOS + PB@QUE + NIR groups, further implying the anti-senescent effects of the hydrogel coatings (Fig. 5C).

These results indicated that SDF-1α, PB, and QUE all functioned as effective anti-senescence components for BMSCs within the hydrogel coatings. Decreased levels of SDF-1α have been reported to correlate with age-related bone loss [58]. Accordingly, supplementation of SDF-1α has been proposed as a potential strategy for delaying cellular senescence, which was further supported by the present findings. Besides, QUE possesses multiple biological activities, such as anti-oxidant, anti-inflammatory, and anti-apoptosis effects. Among these functions, the antioxidant property of QUE has been identified as particularly important for alleviating age-related musculoskeletal disorders [59]. Consistently, SASP expression levels in the ATOS + PB@QUE and ATOS + PB@QUE + NIR groups were obviously reduced compared with those in the other groups, indicating the pronounced anti-senescent effects of QUE. PB nanoparticles exhibit high redox potential and can readily undergo oxidation–reduction transitions [60], endowing them with potent anti-oxidant activities. Previous studies have demonstrated that PB could inhibit SASP secretion in ultraviolet A-induced senescent human dermal fibroblasts [61]. Overall, the constituent components incorporated into the hydrogel coatings synergistically exerted anti-senescence effects, thereby contributing to the attenuation of the SME.

Furthermore, the potential mechanisms of the hydrogel coatings on delaying the senescence of BMSCs were investigated from the perspectives of autophagy. The p62 expression, an autophagy adaptor protein, gradually decreased in the ATO, ATOS, ATOS + PB, and ATOS + PB@QUE groups, with the p62 fluorescence intensity declining from 40515.33 ± 4404.48 in the ATO group to 8583.33 ± 495.94 in the ATOS + PB@QUE group (Fig. 5E and F). Meanwhile, the fluorescence intensity of LC3B exhibited a correspondingly increasing trend, increasing from 4363.67 ± 512.19 in the ATO group to 34892.67 ± 2554.87 in the ATOS + PB@QUE group (Fig. 5G and H). However, no significant differences in p62 and LC3B expression were observed between the ATOS + PB@QUE and ATOS + PB@QUE + NIR groups. These results indicated that the hydrogel coatings promoted autophagy, which represents a key mechanism for the clearance of senescent cells [62]. SDF-1α has been reported to contribute to autophagy induction and to stimulate chondrocyte autophagy through the CXCR4/mammalian target of rapamycin (mTOR) signaling pathway [63]. Similarly, QUE has been found to activate autophagy and exert protective effects against cellular damage [64]. Accordingly, SDF-1α and QUE were incorporated into the hydrogel coatings to activate autophagy to remove senescent cells. Notably, NIR addition did not lead to further attenuation of senescence or enhancement of autophagy, which may be attributed to the irreversible nature of cellular senescence and the stable phenotypic alterations that are not easily reversed by transient NIR stimulation [65].

### 2.7 Osteogenic differentiation of BMSCs in different groups

**Fig. 6:**
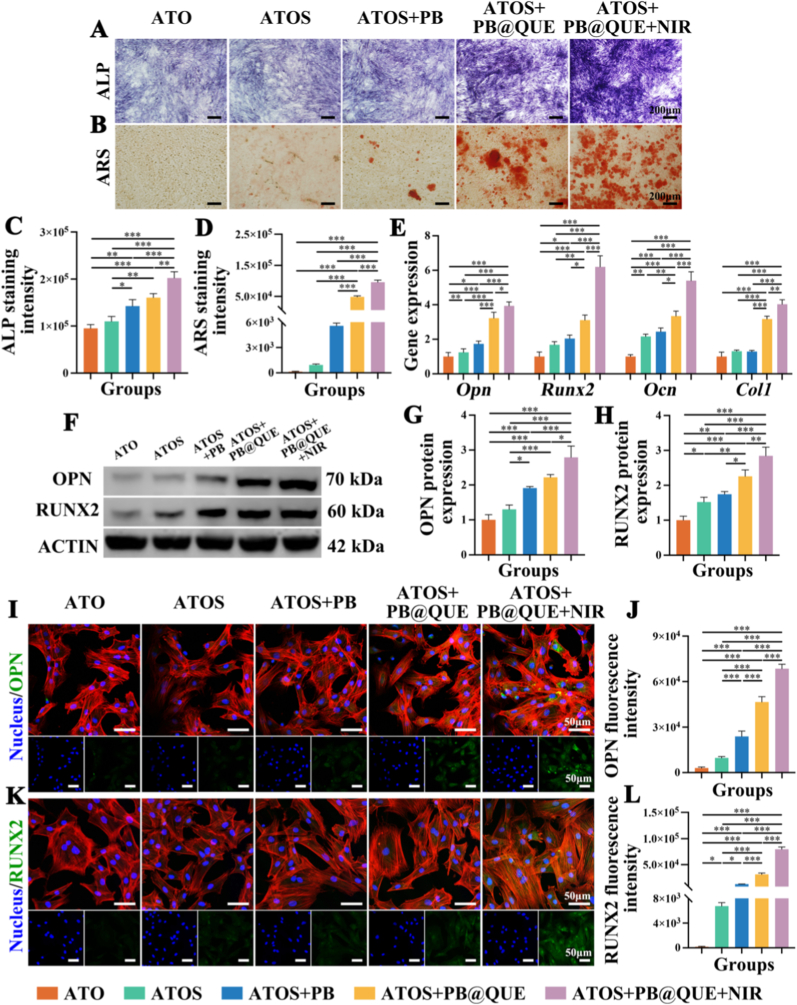
Osteogenic differentiation of BMSCs in different groups. (A, B) Representative images of (A) ALP and (B) ARS staining. (C, D) Quantitative analysis of (C) ALP and (D) ARS staining intensity. (E) mRNA expression levels of *Opn*, *Runx2*, *Ocn,* and *ColI* in BMSCs. (F) Protein expression levels of OPN and RUNX2 by Western Blotting. (G, H) Quantitative analysis of (G) OPN and (H) RUNX2 protein expression. (J, K) Representative fluorescence images of (I) OPN and (K) RUNX2 staining. (J, L) Quantitative analysis of (J) OPN and (L) RUNX2 fluorescence intensity. ∗ *p* < 0.05, ∗∗ *p* < 0.01, and ∗∗∗ *p* < 0.001, n = 3.

First, the migration ability of BMSCs was evaluated using scratch assays and transwell assays. The results demonstrated that BMSCs in the ATOS group migrated faster than those in the ATO group (Figs. S12 and S13), suggesting that SDF-1α effectively enhanced the migration of BMSCs. Then, alkaline phosphatase (ALP) staining and alizarin red S (ARS) staining were performed to evaluate the osteogenic differentiation of BMSCs under different treatments. The staining results indicated that the hydrogel coatings markedly increased the intensity of ALP and ARS in BMSCs, with the strongest effects observed in the ATOS + PB@QUE + NIR groups, followed by the ATOS + PB@QUE, ATOS + PB, and ATOS groups (Fig. 6A–D). Meanwhile, the expression levels of osteogeneis-related genes were analyzed. The results showed the mRNA expression levels of *Opn*, *Runx2*, *Ocn,* and *Col1* were higher in the ATOS + PB@QUE and ATOS + PB@QUE + NIR groups than in the other groups (Fig. 6E). Also, western blot analysis revealed that the protein levels of OPN and RUNX2 were significantly upregulated in the ATOS + PB@QUE + NIR group (Fig. 6F–H). Immunofluorescence staining further confirmed these findings. The fluorescence intensity of OPN increased from 3045.00 ± 679.02 in the ATO group to 68497.33 ± 2965.52 in the ATOS + PB@QUE + NIR group, which exhibited the highest level among all groups (Fig. 6I–J). Similarly, RUNX2 immunofluorescence staining showed a comparable trend, with the highest fluorescence intensity observed in the ATOS + PB@QUE + NIR group, followed by the ATOS + PB@QUE, ATOS + PB, ATOS, and ATO groups (Fig. 6K–L). Overall, the hydrogel coatings exhibited brilliant bone induction activity. Among the components of the hydrogel coatings, SDF-1α is a key chemotactic factor for BMSCs and plays an essential role in regulating BMSC migration [66]. Previous studies have demonstrated that SDF-1α promotes osteogenic differentiation of BMSCs via Janus kinase 2 (JAK2)/signal transducer and activator of transcription 3 (STAT3) pathway [67]. Moreover, QUE has been reported to promote osteogenesis of BMSCs by suppressing repetitive element-triggered RNA-sensing pathways [68], and it has been widely regarded as a common constituent of biomaterials with osteogenic activities [69]. In addition, SDF-1α has been shown to enhance autophagy and promote the clearance of senescent BMSCs, while simultaneously facilitating cell recruitment and delaying senescence, which may collectively contribute to its osteogenic effects. Therefore, SDF-1α and QUE were incorporated into the hydrogel coatings to synergistically enhance osteogenic potential. Notably, the ATOS + PB@QUE + NIR group showed superior osteogenic activity compared with the ATOS + PB@QUE group, suggesting that moderate hyperthermia is conducive to osteogenesis [62]. NIR was found to upregulate heat shock proteins, thereby accelerating mineralization, enhancing collagen stability, and increasing the expression of osteogenic markers. Meanwhile, NIR can promote ubiquitination and degradation of cryptochrome 1 (CRY1), leading to activation of the bone morphogenetic protein (BMP) signaling pathway and further enhancement of osteogenic differentiation [70]. Therefore, the ATOS + PB@QUE + NIR group showed the highest expression levels of osteogenesis-related genes, suggesting the most effective osteogenetic induction among all groups.

### 2.8 The effects of the hydrogel coatings on macrophage polarization

**Fig. 7:**
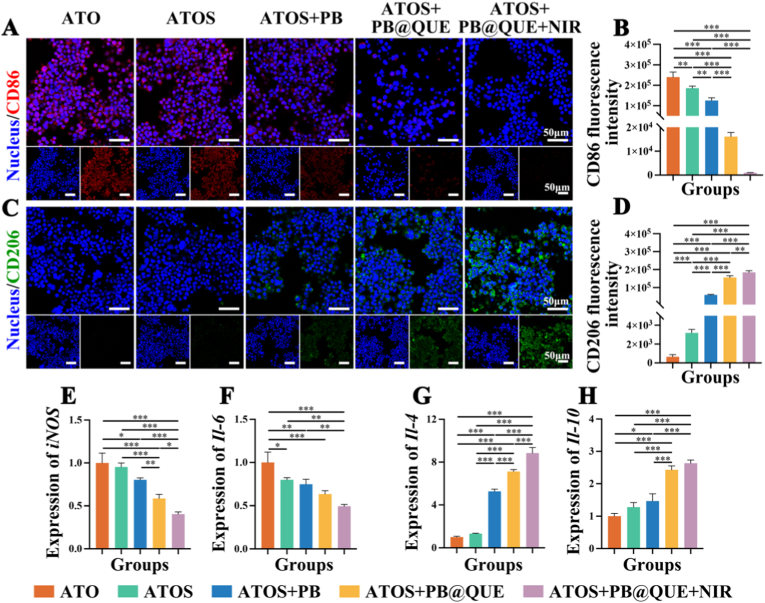
Effects of different treatments on macrophage polarization of RAW264.7 cells. (A) Representative fluorescence images of CD86 staining. (B) Quantitative analysis of CD86 fluorescence intensity. (C) Representative fluorescence images of CD206 staining. (D) Quantitative analysis of CD206 fluorescence intensity. (E–H) mRNA expression levels of *iNOS*, *Il-6*, *Il-4,* and *Il-10* in RAW264.7 cells. ∗ *p* < 0.05, ∗∗ *p* < 0.01, and ∗∗∗ *p* < 0.001, n = 3.

Macrophages play key roles in the senescent microenvironment by regulating immune responses. Accordingly, macrophage polarization was further explored. The M1 macrophage marker CD86 and the M2 macrophage marker CD206 were detected by immunofluorescence staining [71,72]. The results found that the four groups (ATOS, ATOS + PB, ATOS + PB@QUE, and ATOS + PB@QUE + NIR) exhibited lower CD86 fluorescence intensity and higher CD206 fluorescence intensity compared with the ATO group (Fig. 7A–D). Meanwhile, a progressive reduction in CD86 expression accompanied by a concomitant increase in CD206 expression was observed across the ATOS, ATOS + PB, ATOS + PB@QUE, and ATOS + PB@QUE + NIR groups (Fig. 7A–D). Among these groups, the ATOS + PB@QUE + NIR group exhibited the lowest CD86 intensity and highest CD206 intensity, suggesting that polarization of macrophages toward the M2 anti-inflammatory phenotype (Fig. 7A–D). In parallel, the expression levels of inflammatory cytokines were further examined. The results revealed that the expression of pro-inflammatory cytokines (*iNOS* and *Il-6*) was markedly decreased, whereas the expression of anti-inflammatory cytokines (*Il-4* and *Il-10*) was increased following treatment with the hydrogel coatings (Fig. 7E–H). When exposed to lipopolysaccharide (LPS), resting macrophages (M0) are known to polarize toward a pro-inflammatory M1-like phenotype and release pro-inflammatory cytokines [64]. In contrast, M2 macrophages facilitate debris clearance and tissue repair by the secretion of anti-inflammatory cytokines, such as IL-10 and IL-4 [73,74]. In this study, the hydrogel coatings effectively directed macrophages polarization toward the M2 anti-inflammatory phenotype, thereby supporting tissue repair. Previous studies have reported that QUE inhibits inflammatory responses and promotes diabetic wound healing by inducing macrophage polarization from the M1 to the M2 phenotype [75,76]. Similarly, PB has also found to attenuate LPS-stimulated inflammation in macrophages and to promote M2 macrophage polarization [77]. PB nanoparticles exhibit excellent hydroxyl radical affinity and function as ROS scavengers, mimicking the activities of antioxidant enzymes such as peroxidase, catalase, and superoxide dismutase [78]. By scavenging ROS and inactivating NF-κB/mitogen-activated protein kinase (MAPK) signaling pathways, PB nanoparticles have been reported to inhibit osteoclastogenesis and modulate inflammatory responses. The incorporation of PB reduces ROS levels, thereby promoting macrophage polarization toward the M2 phenotype [79]. On this basis, the mild photothermal effect generated by NIR further enhanced M2-type macrophage polarization. The combination of PB and NIR further promoted M2 macrophage polarization and enhanced the anti-inflammatory effects of the hydrogel coatings [80,81]. In addition, Luo et al. proved that QUE possessed protective effects on macrophage pyroptosis via regulation of the Toll-like receptor 2 (TLR2)/myeloid differentiation factor 88 (Myd88)/NF-κB and ROS/AMP-activated protein kinase (AMPK) signaling pathway [82]. Taken together, these findings indicate that the hydrogel coatings incorporating QUE and PB, in combination with NIR stimulation, exhibit strong regulatory effects on macrophage phenotype transition.

### 2.9 The effects of titanium scaffolds with hydrogel coatings in vivo

**Fig. 8:**
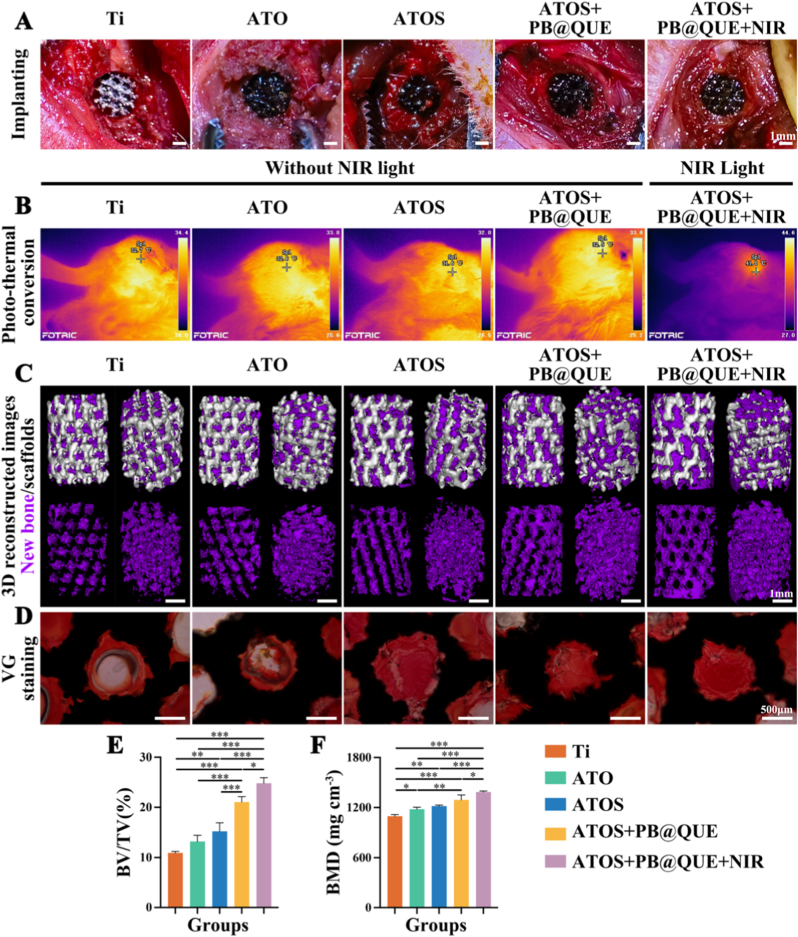
In vivo performance of hydrogel-coated scaffolds in an osteoporotic rat model. (A) Surgical implantation of scaffolds into the femoral condyle of rats with OVX. (B) Infrared thermographic images showing photothermal conversion. (C) Evaluation of newly formed bone by micro-CT. (D) VG staining for collagen fiber. Quantitative analysis of (E) BV/TV and (F) BMD obtained from micro-CT results. ∗ *p* < 0.05, ∗∗ *p* < 0.01, and ∗∗∗ *p* < 0.001, n = 3.

Furthermore, the in vivo effects of titanium scaffolds with hydrogel coatings were investigated. Osteoporotic rat models were constructed by ovariectomy (OVX) according to a previous method [83]. Micro-CT analysis of femurs suggested the successful construction of OVX models (Fig. S14). Subsequently, scaffolds from the different groups were implanted into the femoral condyle of rats with OVX (Fig. 8A). Post-implantation infrared thermal imaging analysis showed that the ATOS + PB@QUE + NIR group reached the preset temperature of 41.0 °C in the rat model (Fig. 8B). The volume of newly formed bone in each group was quantified by micro-CT analysis. The results indicated that the ATOS + PB@QUE + NIR group exhibited the most pronounced increase in new bone volume compared with the other groups (Fig. 8C). Quantitative analyzing of the bone volume/tissue volume (BV/TV) and bone mineral density (BMD) further supported these observations. Among all groups, the ATOS + PB@QUE + NIR group ranked highest, followed by the ATOS + PB@QUE, ATOS, ATO, and Ti groups (Fig. 8E and F). Specifically, the BV/TV values were 10.94 ± 0.31 % for the Ti group, 13.22 ± 1.23 % for the ATO group, 15.23 ± 1.72 % for the ATOS group, 21.07 ± 1.08 % for the ATOS + PB@QUE group, and 24.80 ± 1.14 % for the ATOS + PB@QUE + NIR group. Similarly, the BMD values were 1096.46 ± 18.46 mg g^−3^ for the Ti group, 1179.46 ± 23.78 mg g^−3^ for the ATO group, 1217.35 ± 11.48 mg g^−3^ for the ATOS group, 1292.22 ± 58.79 mg g^−3^ for the ATOS + PB@QUE group, and 1386.86 ± 12.39 mg g^−3^ for the ATOS + PB@QUE + NIR group. The collagen fiber content was further evaluated *via* Van Gieson (VG) staining. Consistent with the micro-CT results, the ATOS + PB@QUE + NIR group exhibited the highest collagen content among all groups (Fig. 8D). In addition, VG staining revealed a gradual increase in collagen content at the bone-implant interface from the Ti group to the ATOS + PB@QUE + NIR group (Fig. S15). Additionally, the biological toxicity of the different groups in vivo was also evaluated. Hematoxylin-eosin (HE) staining of major organs showed no abnormal pathological changes, indicating favorable biosafety of the implanted materials (Fig. S16). Osteoporotic bone defects are highly prevalent in the elderly, highlighting the urgent need for effective therapeutic strategies. Meanwhile, defects of the medial femoral condyle represent a commonly used and clinically relevant model for bone defect repair [84]. Thus, a medial femoral condyle defect model was established in osteoporotic rats to investigate the effects of the composite scaffolds in vivo. New bone formation was assessed from multiple perspectives, including bone volume, bone mineral density, and collagen fiber deposition. Consistent with expectations, the titanium scaffolds coated with multifunctional hydrogels displayed superior bone regeneration capability and favorable biocompatibility [85]. SDF-1α plays a critical role in activating autophagy, delaying cellular senescence, and promoting the recruitment and osteogenic differentiation of BMSCs [86]. QUE acts as an anti-senescence enhancer by promoting autophagy, facilitating osteogenic differentiation of BMSCs, and regulating macrophage polarization. PB, owing to its excellent anti-oxidant properties, has shown effects in slowing down aging, inducing osteogenesis, and promoting M2 macrophage polarization. Notably, the photothermal effects induced by NIR further amplify the biological functions of PB, including the promotion of BMSC osteogenesis and the regulation of macrophage polarization.

In this study, to facilitate hard-tissue sectioning, staining, and visualization of new bone within the scaffold, the entire scaffold was intactly removed from the bone defect. However, this approach limits direct evaluation of bone integration at the scaffold–host interface. Future studies will systematically investigate the efficiency of osseointegration at the bone–implant interface to enhance the clinical relevance of this system.

## 3 Conclusion

In this study, a photothermal-responsive and bioactive hydrogel coating integrated with a drug delivery system (ATOS + PB@QUE + NIR) was successfully constructed on the titanium alloy scaffolds. This multifunctional hydrogel coating effectively regulates the biotribological properties at the bone-tissue interface and enables controlled drug release at the bone defect site, thereby suppressing inflammation, scavenging ROS, delaying cellular senescence, promoting osteogenic differentiation of BMSCs, and modulating macrophage polarization. Through the synergistic regulation of these biological processes, the hydrogel-coated titanium scaffolds significantly accelerate the repair of osteoporotic bone defects. Overall, this research provides an effective strategy for personalized and precise treatment of osteoporotic bone defects and demonstrates substantial potential for future clinical and translational applications.

## 4 Methods

### 4.1 Reagents and materials

Lithium Phenyl (2,4,6-trimethylbenzoyl) phosphinate (LAP, 97 %) and AAPBA(97 %) were purchased from Haohong Biomedical Technology Co., Ltd. (Shanghai, China). (3-Aminopropyl) triethoxysilane (ATPES, 98 %), dopamine (PDA, 99 %+), TA, and MBAA (>99 %) were obtained from Shanghai Titan Scientific Co., Ltd. (Shanghai, China). AM (≥99.0 %) was purchased from Shanghai Discovery Technology Co., Ltd. (Shanghai, China). Hollow PB nanoparticles were obtained from KeyGEN Biotech Co., Ltd. (Nanjing, China). SDF-1α/CXCL12 protein, OGP (98.35 %), and QUE (98.45 %) were purchased from MedChemExpress (USA). A live/dead cell double-staining kit (KGA9501-100) was obtained from KeyGENBiotech (Nanjing, China), and a CCK-8 assay kit (TM772) was purchased from Dojindo (Tokyo, Japan). Primary antibodies against OPN (22952-1-AP), RUNX2 (20700-1-AP), CD86 (13395-1-AP), CD206 (18704–1-AP), p21 (10355-1-AP), p16 (10883-1-AP), LC3B (14600-1-AP), and CL594-phalloidin (PF00003) were obtained from Proteintech (Wuhan, China). Secondary antibodies against anti-rabbit IgG (H + L), F(ab')2 Fragment (Alexa Fluor® 488 Conjugate) (4412), anti-rabbit IgG (H + L), F(ab')2 Fragment (Alexa Fluor® 594 Conjugate) (8889), anti-rabbit IgG, HRP-linked antibody (7074), β-Actin (13E5) rabbit mAb (4970) were obtained from Cell Signaling Technology (CST, Boston, USA). The EZBioscience EZ-press RNA purification kit was obtained from EZBioscience (Suzhou, China). PrimeScript™ RT Master Mix and TB Green Premix Ex Taq II were purchased from Takara (Japan). The SA-β-Gal staining kit, BCIP/NBT Alkaline Phosphatase Color Development Kit, and Alizarin Red S Staining Kit were obtained from Beyotime (Shanghai, China). Osteogenic induction medium was obtained from OriCell (Guangzhou, China). All reagents for cell culture were purchased from Gibco (Carlsbad, USA).

Medical-grade Ti6Al4V cuboids (20 mm × 20 mm × 2 mm) with a surface roughness of 0.10 ± 0.02 μm were selected for tribological tests and purchased from Baoji Junhang Metal Materials Co., Ltd., and their elemental compositions are shown in Table S1. 3D-printed Ti6Al4V scaffolds (diameter: 3.0 mm; height: 4.4 mm; pore size: ∼100 μm) were used for biological and in vivo experiments. SBF (pH 7.4–7.6) was purchased from Shanxi Zhonghui Hemocai Biomedical Technology Co., Ltd. (Shanxi, China), and its composition is listed in Table S2. The upper friction pairs used in tribological tests were cortical bone sphere samples with a diameter of 6.35 mm, all collected from the femurs of adult bulls (under the articular cartilage) and stored at 2–8 °C. Their surface roughness was 8.256 ± 0.037 μm. Tribological tests, animal experiments, and cell experiments were performed using an 808 nm NIR laser system (FC-808-5000-MM, Changchun Laser Technology Co., Ltd., China). The laser output power was adjusted to maintain a constant temperature of 41 ± 0.5 °C, monitored in real time using an infrared thermal imaging camera (Model FOTRIC 625C, Shanghai Thermal Imaging Technology Co., Ltd., China) with a spatial resolution of 320 × 240 pixels. The laser beam was focused to a spot diameter of approximately 10 mm at the target surface to ensure complete coverage of the irradiated area. Each sample was irradiated continuously for 10 min under these conditions.

### 4.2 Preparation of PB@QUE-Incorporated hydrogel coatings

5 mL of PB solution (40 μg/mL) was mixed with 2 mg of QUE and stirred at 1000 rpm for 12 h. The mixture was centrifuged at 11000 rpm, and the precipitate was resuspended in phosphate-buffered saline (PBS) to a final volume of 5 mL to obtain quercetin-loaded Prussian blue (PB@QUE). A prepolymer solution containing 40 % W/V AM, 0.2 % W/V AAPBA, 1 % W/V TA, 0.1 % W/V MBAA, 20 μg/mL OGP, and 0.2 % W/V LAP was prepared in 5 mL PBS. The mixture was vacuum-stirred for 30 min to remove air bubbles, followed by sonication for 30 min. Subsequently, the prepolymer solution was transferred into a mold and irradiated under 365 nm UV light for 10 min to initiate polymerization, yielding the ATO hydrogel. To prepare the ATO + PB@QUE composite hydrogel, 40 μg/mL PB@QUE was additionally incorporated into the above prepolymer solution. After sonication, the mixture was poured into a mold and subjected to UV-initiated polymerization to obtain the ATO + PB@QUE hydrogel. For preparation of the ATO + PB hydrogel, 40 μg/mL PB was added to the prepolymer solution following the same procedure. Following the above hydrogel preparation protocol, 100 ng/mL of SDF-1α was added to the prepolymer solution to prepare hydrogels for cell experiments, yielding the ATOS, ATOS + PB, and ATOS + PB@QUE hydrogels. The detailed experimental groups are summarized in Table S3.

Ti6Al4V plates and scaffolds were pre-modified by grafting APTES and DA according to previous reported methods to enhance surface adhesion [87,88]. The modified Ti6Al4V substrates were placed in a vacuum spin coater, and the prepolymer solution was uniformly applied to the substrate surfaces via spin coating. Specifically, 1 mL of the prepolymer solution was applied onto each sample, followed by spin coating at 50 rpm for 10 s and then at 300 rpm for 20 s. This spin-coating procedure was repeated 20 times for each sample group. Subsequently, the coated substrates were irradiated with UV light for 10 min under a nitrogen atmosphere to complete curing of the hydrogel coating. Finally, the resulting samples were immersed in PBS to remove unreacted monomers, yielding the final hydrogel-coated titanium alloy substrates. Square titanium alloy plates were used for tribological performance evaluation, whereas Ti6Al4V scaffolds were employed for in vivo implantation experiments. ATO and ATO + PB coatings were applied to square Ti6Al4V plates for biological tribological tests. 3D-printed Ti6Al4V scaffolds were coated with ATOS, ATOS + PB, and ATOS + PB@QUE for in vivo implantation to evaluate biocompatibility, stability, and therapeutic efficacy.

### 4.3 Structural characterization

FTIR spectroscopy (EQUINOX 55, Thermo Fisher Scientific) was employed to systematically analyze the chemical structures of PB, QUE, and PB@QUE powders, as well as those of the ATO, ATO + PB, and ATO + PB@QUE hydrogels. Raman spectra of PB, QUE, and PB@QUE solutions were recorded using a laser Raman spectrometer (BWS415-532H, B&W Tek, USA) with a 532 nm excitation under dark conditions at room temperature. UV-visible spectroscopy was used to characterize the UV absorption properties of PB, QUE, and PB@QUE, allowing identification of their respective characteristic absorption peaks. A standard calibration curve for QUE concentration was constructed by measuring the absorbance of a series of QUE standard solutions with known concentrations at the characteristic peak of 378 nm. For QUE release experiments, the absorbance of the release medium containing PB@QUE in PBS was measured using a UV-vis spectrophotometer. Specifically, 5 mL of PB@QUE solution was prepared, centrifuged, and resuspended in PBS, followed by continuous stirring at room temperature. The real-time concentration of QUE was calculated using the standard curve, and the cumulative release rate was subsequently determined. The cumulative release percentage was calculated using the following formula:

(1) $$\text{Cumulative}\;\text{release}\;\left(\%\right)=\frac{\text{Cumulative}\;\text{release}\;\text{amount}\;\text{at}\;\text{time}\;t}{\text{Initial}\;\text{total}\;\text{load}\;\text{amount}}\times 100\;\%$$

The ATO and ATO + PB hydrogels were dried at 70 °C for 6 h to remove residual moisture. Subsequently, approximately 15 mg of each dried sample was accurately weighed and placed in alumina crucibles. DSC measurements were performed using a DSC 3 instrument (Mettler-Toledo, Switzerland) under a high-purity nitrogen atmosphere (flow rate: 50 mL min^−1^) at a heating rate of 10 °C min^−1^ from 25 °C to 600 °C to investigate melting-crystallization behavior and glass transition properties. TGA was conducted using a TGA 2 instrument (Mettler-Toledo, Switzerland) to evaluate the thermal stability of the samples.

TEM characterization of PB and PB@QUE was conducted using a FEI Titan Krios G3i microscope, operated at an accelerating voltage of 80–300 kV with an information resolution of 0.14 nm. Elemental mapping images of C, N, O, and Fe were collected simultaneously. The topological structures of the ATO and ATO + PB hydrogels were observed using a scanning electron microscope (SEM, Nova 450, FEI, USA), and porosity was analyzed and calculated using ImageJ software (National Institutes of Health, Bethesda, MD, USA). XRD analysis was performed using a D8 DaVinci diffractometer (Bruker) with Cu Kα radiation (λ = 1.5406 Å) to characterize the crystalline microstructures of the ATO and ATO + PB hydrogels.

The ATO and ATO + PB hydrogel coating samples dried for 24 h were weighed to obtain their initial dry weights (Wa). The samples were then immersed in 30 mL of PBS (pH = 7.4) at 37 °C. At 2 h intervals, surface-adsorbed water was gently removed using filter paper, and the swollen weights (Wb) were recorded. The swelling ratio (SR) was calculated using the following formula (2):

(2) $$S_{R}=\left(W_{b}\;‐\;W_{a}\right)/\;W_{a}\times 100\;\%$$

All experiments were repeated at least three times, and the results are presented as the mean ± standard deviation.

### 4.4 Mechanical property testing

The dynamic rheological properties of ATO and ATO + PB hydrogels were characterized using an MCR301 rheometer (Anton Paar, Austria). All measurements were performed at 25 °C with a fixed frequency of 1 Hz, and the instrument gap was set to 1.0 mm. Strain sweep tests were performed from 0.001 % to 1000 % to determine the linear viscoelastic region of the samples. Subsequently, frequency sweep tests were carried out in the range of 0.1 Hz–25 Hz under a constant strain of 1 % to obtain the viscoelastic moduli. To verify the dynamic reversibility of the hydrogels, alternating step strain tests were performed under low strain (0.1 %) and high strain (10 %) conditions. Additionally, the temperature-dependent rheological behavior of the two hydrogels was examined by monitoring stress variations from 25 °C to 60 °C under a frequency of 1 Hz and a strain of 1 %.

The tensile and compressive properties of the two hydrogels were tested using a CMT6103 electronic universal testing machine (MTS Systems Corporation, USA). For tensile testing, hydrogel samples were molded into rectangular shapes with dimensions of 10 mm × 10 mm × 15 mm (length × width × height). Young's modulus and toughness were calculated from the slope of the linear viscoelastic region in the stress-strain curves. Besides, cyclic compression tests of ATO and ATO + PB hydrogels were performed within the maximum strain range determined from the compressive strain tests. For compression testing, hydrogel samples were molded into cylindrical specimens with a diameter of 12 mm and a height of 10 mm. All compression tests were conducted at a loading rate of 5 mm min^−1^ and included two test modes: compressive strain tests and cyclic loading tests. The interfacial adhesion strength between the Ti6Al4V substrate and the hydrogel coating was tested using the scratch mode of a UMT Tribolab (Bruker, USA). The experimental setup included a 50 N load cell, with a loading rate set at 20 N min^−1^ and a scratch velocity maintained at 4 mm min^−1^. All mechanical tests were replicated at least three times to ensure data reproducibility.

### 4.5 Tribological tests and electrochemical tests

Prior to testing, all titanium plates and 3D-printed scaffolds were cleaned with acetone for 30 min, then rinsed with deionized water three times and dried naturally. The samples were then ultrasonically cleaned in a 20 vol % dilute nitric acid solution for 30 min using an ultrasonic cleaner, and subsequently ultrasonically rinsed with deionized water for another 30 min to remove oil contaminants. The cleaned titanium alloy plates and scaffolds were directly used as control groups. For the coating groups, Ti plates and scaffolds were treated with ATPES and PDA before subsequent coating procedures.

Reciprocating tribological experiments were conducted using a UMT Tribolab tribometer (Bruker, USA) under the lubrication with SBF. Bone balls with a diameter of 6.35 mm were paired with coated Ti6Al4V plates to simulate soft-hard contact at the bone-implant interface. The tribological tests were performed under the following conditions: normal load of 1 N, frequency of 1 Hz, stroke length of 2 mm, and test duration of 30 min. Each group of samples was tested at least three times. The tests yielded COF curves, from which the ACOFwas calculated. After the tribological tests, a 3D white light interferometer (Contour GT-K0, Bruker, USA; WLI) was used to record the wear morphologies of both the upper and lower friction pairs. The wear rate (k) was calculated according to the following formula (3):

(3) $$k=\frac{V_{all}}{X\cdot F_{n}}$$

where V_all_ represents the total wear volume of the bone ball and coating, X denotes the sliding distance, and F_n_ is the normal load. Each sample was tested in triplicate or more to obtain reliable test data. In addition, the thicknesses of the ATO and ATO + PB coatings on the titanium alloy substrate were also measured using a 3D white light interferometer.

Meanwhile, the wear surface morphologies of all friction pairs were observed using SEM. Elemental analysis was performed using a Nova NanoSEM 230 energy-dispersive spectrometer (EDS, FEI, USA) with a resolution of 125 eV (Mn Kα) to verify the wear mechanism.

### 4.6 Cell counting Kit-8 test

The hydrogels were immersed in alcohol for 3 min and washed with PBS three times, then the hydrogels were subjected to UV sterilization for 10 min on one side, flipped over, and sterilized for an additional 10 min on the opposite side [[89], [90], [91]]. Hydrogel extracts were then prepared according to the previous method [92]. Specimens from different groups were immersed in 10 mL of complete culture medium and incubated at 37 °C for 30 min to allow initial swelling. After this period, the supernatant was removed and replaced with 50 mL of fresh culture medium. The hydrogel coatings were then incubated for an additional 24 h to obtain the corresponding extracts for subsequent experiments. The cytocompatibility of the hydrogel coatings was evaluated by assessing their effects on the proliferation of BMSCs using a CCK-8 assay. BMSCs were seeded into 24-well plates at a density of 5 × 10^4^ cells per well in α-MEM medium supplemented with 10 % fetal bovine serum (FBS) and 1 % Penicillin-Streptomycin(PS). After 24h of incubation, hydrogels from different groups or their corresponding extracts were added directly to each well for co-culture. Cell proliferation was assessed on days 1, 4, and 7. At each time point, 500 μL of CCK-8 working solution (prepared by mixing serum-free α-MEM and CCK-8 reagent at a ratio of 9:1) was added to each well, followed by incubation at 37 °C for 2 h. Subsequently, 100 μL of the supernatant from each well was transferred to a 96-well plate, and the absorbance was measured at 450 nm using a microplate reader (Tecan, Switzerland).

### 4.7 Cell live/dead staining

A Live/Dead cell double-staining kit was used to investigate the cytotoxicity of the hydrogel coatings. BMSCs were seeded on the confocal plates at a density of 2 × 10^4^ cells per plate and cultured. After 24 h of incubation, hydrogels from different groups or their corresponding extracts were added for co-culture for an additional 1 day. Subsequently, 500 μL Live/Dead working solution, prepared in α-MEM medium containing AM (1: 1000) and PI (1:2000), was added to each sample and incubated for 30 min. Live cells were stained with AM and exhibited green fluorescence, whereas dead cells were stained with PI and exhibited red fluorescence. The images were acquired by a confocal microscope (Leica, Germany). The number of live and dead cells was counted, and the ratio of live cells and total cells was calculated.

### 4.8 Cell adhesion test

The adhesion behavior between BMSCs and hydrogel coatings was evaluated using confocal microscopy. Hydrogels from different groups were placed in confocal dishes before cell seeding. BMSCs were prepared as a cell suspension at a density of 2 × 10^5^ cells mL^−1^, and 100 μL of the cell suspension was carefully seeded onto the hydrogel surface of each hydrogel sample. The samples were incubated at 37 °C in a humidified atmosphere of 5 % CO_2_ for 30 min to allow initial cell attachment, followed by the addition of 2 mL of complete medium for further culture for 3 days. After incubation, the culture medium was removed, and the samples were gently rinsed three times with PBS at 5 min intervals. Cells were then fixed with 4 % paraformaldehyde for 10 min and washed three times with PBS. F-actin was labeled using CoraLite®594-conjugated Phalloidin (1:500) for 1 h, and cell nuclei were stained with 4′,6-diamidino-2-phenylindole (DAPI) for 10 min. After staining, cell morphology and adhesion were observed using a confocal microscope. Quantitative analysis of actin and cell coverage was performed using ImageJ software.

### 4.9 Senescence-associated *β*-galactosidase staining

*β*-galactosidase is the specific marker of cell senescence and is considered the gold standard for senescence tests. BMSCs were seeded on 24-well plates at a density of 5 × 10^4^ per well and cultured in α-MEM medium with 10 % FBS and 1 % PS for 24 h. A cellular senescence model was established by treating BMSCs with hydrogen peroxide (H_2_O_2_, 200 μM) for 24 h. Meanwhile, hydrogels from different groups were introduced and co-cultured with the cells under standard conditions (37 °C, 5 % CO_2_). After 1 day of treatment, the culture medium was removed, and the cells were fixed using 1 mL of *β*-galactosidase fixation solution at room temperature for 15 min. Following fixation, the solution was discarded, and the cells were rinsed three times with PBS (5 min per wash). Subsequently, 1 mL of freshly prepared *β*-galactosidase staining solution was added to each well, and the samples were incubated overnight at 37 °C in a non-CO_2_ incubator. Stained cells were observed using a light microscope (Olympus, Japan). Quantitative analysis of SA-*β*-gal staining intensity was performed using Image Pro Plus 6.0 software (Media Cybernetics, Rockville, MD, USA).

### 4.10 RT-qPCR test

For osteogenic induction, the culture medium of BMSCs was replaced with osteogenic induction medium and maintained for an additional 7 days. For senescence induction, BMSCs were treated with medium containing H_2_O_2_ for 24 h. For macrophage activation, cells were treated with lipopolysaccharide (LPS, 100 ng mL^−1^) for 24h. After induction, hydrogels from different groups were added, and the cells were maintained for an additional 24 h. Total RNA was then extracted and purified using the EZ-press RNA Purification Kit (EZBioscience, USA) according to the manufacturer's protocol. Reverse transcription was performed with 5X PrimeScript RT Master Mix for qPCR (Takara, Japan). RT-qPCR was carried out using TB Green Premix Ex Taq II (Takara, Japan) on a LightCycler 480 system (Roche, Switzerland). The thermal cycling conditions were set according to the manufacturer's protocol. Relative mRNA expression levels were calculated using the 2^−ΔΔCt^ method, with *Gapdh* serving as the internal reference gene. Primer sequences used for RT-qPCR are listed in Table S4.

### 4.11 Immunofluorescence staining of BMSCs

After induction of BMSC senescence by H_2_O_2_ for 24 h and subsequent treatment with hydrogels from different groups for another 24 h, immunofluorescence staining of p21, p62 and LC3B was performed. In brief, the culture medium was removed, and the cells were fixed with 4 % paraformaldehyde for 30 min at room temperature. After washing with PBS for 3 times, the cells were permeabilized with 0.5 % Triton X-100 and blocked with 5 % BSA sequentially. Primary antibodies were then added, and the samples were incubated overnight at 4 °C. On the following day, the cells were incubated with secondary antibodies, including anti-rabbit IgG (H + L), F(ab')_2_ fragment (Alexa Fluor® 488 conjugate) and anti-rabbit IgG (H + L), F(ab')_2_ fragment (Alexa Fluor® 594 conjugate), both diluted 1:5000, for 1 h at room temperature. Cell nuclei were counterstained with DAPI for 10 min. Fluorescence images were obtained using a confocal microscope. The immunofluorescence intensity was quantified using Image Pro Plus 6.0 software.

### 4.12 ALP staining and quantitative analysis

BMSCs were seeded into 24-well plates at 5 × 10^4^ cells per well and allowed to adhere for 24 h. Different groups were then added for direct co-culture, and the medium was replaced with osteogenic induction medium. The cultures were maintained for 7 days, with medium changes every 2–3 days. On day 7, the cells were fixed in 4 % paraformaldehyde for 10 min at room temperature and rinsed three times with PBS. ALP activity was visualized using a BCIP/NBT ALP Color Development Kit according to the manufacturer's instructions. The staining solution was added to each well and incubated for 30 min in the dark. The reaction was terminated by removing the staining solution and washing the cells three times with PBS. Images were acquired under a light microscope. ALP staining intensity was quantified using Image Pro Plus 6.0 software.

### 4.13 ARS staining and quantitative analysis

To evaluate extracellular matrix mineralization, ARS staining was performed. BMSCs were seeded into 24-well plates at 5 × 10^4^ cells per well and cultured for 24 h. Different groups were then added, and the culture medium was replaced with osteogenic induction medium. After 14 days of osteogenic induction, the culture medium was removed, and the cells were washed with PBS. Cells were fixed with 4 % paraformaldehyde at room temperature for 10 min and washed with PBS. ARS staining solution was added to each well and incubated in the dark for 15 min, followed by three washes with PBS. Stained samples were imaged using an optical microscope, and the fluorescence intensity was quantified using Image Pro Plus 6.0 software.

### 4.14 Western blot analysis

BMSCs were seeded into 6-well plates at a density of 2 × 10^5^ cells per well and cultured for 24 h. Different groups were then added for direct co-culture, and the culture medium was replaced with osteogenic induction medium for continued culture over 7 days. After treatment, cells were lysed using RIPA buffer on ice for 10 min. The lysates were mixed with loading buffer at a ratio of 4:1 and denatured by boiling at 100 °C for 5 min. Protein samples were stored at −20 °C until further use. Protein concentrations were determined using the bicinchoninic acid (BCA) assay according to the manufacturer's instructions. Equal amounts of protein were separated by SDS-PAGE and then transferred onto PVDF membranes using a wet transfer method. The membranes were blocked with non-fat powdered milk at room temperature for 1 h on a shaker. Primary antibodies were incubated with the membranes overnight at 4 °C with the following dilutions: OPN (1:1000), RUNX2 (1:1000), and β-actin (1:1000). After washing, the membranes were incubated with HRP-linked secondary antibody (Anti-rabbit IgG, 1:1000 dilution) for 1 h. Protein bands were visualized using an enhanced chemiluminescence (ECL) substrate, imaged with a chemiluminescence imaging system (eblot), and quantified using ImageJ software.

### 4.15 Osteogenic immunofluorescence staining

BMSCs were seeded into confocal dishes at a density of 2 × 10^4^ cells per well and cultured for 24 h. Different groups were then added for direct co-culture, and the culture medium was replaced with osteogenic induction medium for continued culture for 7 days. After treatment, the cells were fixed with 4 % paraformaldehyde for 30 min at room temperature and washed three times with PBS (5 min per wash) to remove residual fixative. Cells were permeabilized with 0.5 % Triton X-100 and blocked with 5 % BSA. Primary antibodies against OPN (1:500) and RUNX2 (1:500) were added, and the samples were incubated overnight at 4 °C. After washing with PBS, cells were incubated with secondary antibodies (anti-rabbit IgG (H + L), F(ab')_2_ fragment (Alexa Fluor® 488 conjugate), diluted at 1:5000) for 1 h at room temperature. The cytoskeleton was stained with phalloidin for 1 h, and cell nuclei were stained with DAPI for 10 min. Fluorescence images were captured using a confocal microscope, and fluorescence intensity was quantified using ImageJ software.

### 4.16 Immunofluorescence staining of RAW264.7 cells

RAW264.7 cells were seeded into confocal dishes at a density of 2 × 10^4^ cells per well and cultured for 24 h. Different groups were added for direct co-culture, followed by stimulation with LPS for 24 h. Cells were then fixed with 4 % paraformaldehyde for 30 min at room temperature and washed three times with PBS (5 min per wash) to remove residual fixative. Cells were permeabilized with 0.5 % Triton X-100 and blocked with 5 % BSA. Primary antibodies against CD86 (1:500) and CD206 (1:500) were added, and samples were incubated overnight at 4 °C. After washing with PBS, cells were incubated with secondary antibodies (anti-rabbit IgG (H + L), F(ab')_2_ fragment (Alexa Fluor® 488 conjugate) and anti-rabbit IgG (H + L), F(ab')_2_ fragment (Alexa Fluor® 594 conjugate), diluted at 1:5000) for 1 h at room temperature. Cell nuclei were counterstained with DAPI for 10 min. Fluorescence images were obtained using a confocal microscope, and the fluorescence intensity was quantified using Image Pro Plus 6.0 software.

### 4.17 Transwell assay test

The recruitment effect of the materials on BMSCs was evaluated using a Transwell assay. BMSCs were seeded into the upper chambers at a density of 1 × 10^4^ cells per well, while ATO and ATOS hydrogels were placed in the lower chamber. The chambers were incubated at 37 °C with 5 % CO_2_ for 24 h. After incubation, non-migrated cells on the upper surface of the membrane were removed, and the membrane was washed with PBS. Migrated cells were fixed with 4 % paraformaldehyde for 30 min, followed by three washes with PBS. Cells were then stained with 1 mL of crystal violet solution for 30 min, and excess dye was removed by washing with PBS three times. Images were captured using a light microscope, and the positively stained area was quantified using ImageJ software.

### 4.18 Scratch assay test

BMSCs were seeded into six-well plates at a density of 2 × 10^5^ cells per well. After 24 h of incubation, when the cells reached approximately 100 % confluence, a sterile 200 μL pipette tip was used to create a linear scratch perpendicular to the well bottom, guided by a sterile ruler. Detached cells were removed by washing with PBS, and serum-free medium was added. ATO and ATOS hydrogel coatings were introduced, and the cells were cultured for an additional 24 h. The scratch area was observed and imaged under a microscope. Scratch closure was quantified using ImageJ software, and migration rates of each group were calculated.

### 4.19 Animal experiments

All animal experiments were approved by the Animal Ethics Committee of Shanghai JiaoTong University (Study Protocol No: A2024363). Eight-week-old female Sprague–Dawley (SD) rats were used to establish an osteoporotic model by bilateral ovariectomy. Three months after OVX, femora were evaluated by micro-computed tomography (micro-CT, Pingseng Scientific, China) to confirm successful osteoporosis construction. For bone defect surgery, rats were anesthetized, and routine disinfection and draping were performed. A longitudinal skin incision was made along the medial aspect of the femur, and the muscle was bluntly dissected to expose the femoral condyle. A standardized cylindrical bone defect (3 mm diameter and 4.5 mm in depth) was created in the medial femoral condyle under sterile conditions. Scaffolds from different groups were implanted into the defects. For the NIR-treated group, NIR was applied to the defect region once per week for 10 min per session at a power setting of 1 w⋅cm^−2^. Immediately after implantation, thermal imaging tests were conducted at the scaffold-implanted defect sites across different groups. After 8 weeks, femora were harvested for micro-CT analysis using a tube voltage of 80 kV and a current of 60 μA. Then, 3D images were reconstructed, and auxiliary software was used to assess the BV/TV and BMD. Following micro-CT analysis, scaffolds were intactly removed from the femoral condyle for undecalcified hard-tissue sectioning, followed by VG staining. Major organs were also collected and subjected to HE staining to assess systemic biosafety.

### 4.20 Statistical analysis

All data are presented as the mean ± Standard Deviation (SD). Error bars indicate SD. One-way analysis of variance (ANOVA) followed by Tukey's post-hoc analysis was used for comparisons between three or more groups by SPSS version 19.0 (IBM Corporation, USA). Statistical significance was defined at ∗ *p* < 0.05, ∗∗ *p* < 0.01, and ∗∗∗ *p* < 0.001, n = 3.

## Funding

National Natural Science Foundation of China (82301045), Fundamental Research Funds for the Central Universities (24X010301321), Special Science and Technology Projects of the Science and Technology Bureau of Shenyang City Supporting the High-Quality Development of China Medical University (23-506-3-01-24), Natural Science Foundation of Liaoning Provincial (2024-MSLH-539), Collaborative Innovation Fund (XTCX2024-02) from 10.13039/501100008875Shanghai Institute of Technology, Special Fund for Development and Reform of 10.13039/501100008875Shanghai Institute of Technology (101100250066-B03) and sponsored by Collaborative Innovation Center of Fragrance Flavour and Cosmetics.

## CRediT authorship contribution statement

**Chenchen Wang:** Data curation, Funding acquisition, Investigation, Validation, Writing – original draft. **Yuan Wang:** Data curation, Validation, Writing – original draft. **Xiaojun Li:** Data curation, Investigation, Validation, Writing – original draft. **Hao Cao:** Data curation, Investigation. **Chenfeng Wang:** Data curation, Investigation. **Sheng Han:** Data curation, Investigation, Writing – review & editing. **Haotian Chen:** Supervision, Writing – review & editing. **Xin Zhao:** Project administration, Supervision, Writing – review & editing. **Shude Yang:** Data curation, Funding acquisition, Project administration, Supervision, Validation, Writing – original draft, Writing – review & editing.

## Declaration of competing interest

The authors declare that they have no conflicts of interest.

## Data Availability

Data will be made available on request.
