# Supplementary material for: 3D-printed titanium scaffolds coated with a multifunctional photothermal-responsive hydrogel promote osteoporotic bone defect repair
Source: Mater Today Bio. 2026 Jan 29;37:102879. doi: 10.1016/j.mtbio.2026.102879 (PMC12890852; doi:10.1016/j.mtbio.2026.102879)
Supplement: Multimedia component 1 [file mmc1.docx]

3D-printed titanium scaffolds coated with a multifunctional photothermal-responsive hydrogel promote osteoporotic bone defect repair

Chenchen Wang ^a,1^, Yuan Wang ^a,1^, Xiaojun Li ^d,1^, Hao Cao ^a^, Chenfeng Wang ^b,c^, Sheng Han ^a, *^, Haotian Chen ^b,*^, Xin Zhao ^d, *^, Shude Yang ^b,c*^

a Department of Chemical Engineering and Energy Technology, Shanghai Institute of Technology, Shanghai, 201418, China.

b Center of plastic and Cosmetic Surgery, School and Hospital of Stomatology, China Medical University, Liaoning Provincial Key Laboratory of Oral Diseases, Shenyang, Liaoning, 110002, China.

c Department of Plastic Surgery, The First Hospital of China Medical University, Shenyang, Liaoning, 110011, China.

d Shanghai Key Laboratory of Orthopaedic Implants, Department of Orthopaedic Surgery, Shanghai Ninth People’s Hospital, Shanghai Jiao Tong University School of Medicine, No. 639 Zhizaoju Road, Shanghai 200011, China.

^1^ Contributed equally to this work.

^*^ Corresponding author.

E-mail addresses: Shude Yang (sdyang@cmu.edu.cn); Xin Zhao (zhaoxinmlg@126.com); Haotian Chen (18602496184@163.com); Shen Han (hansheng654321@sina.com).

**Supplementary materials**


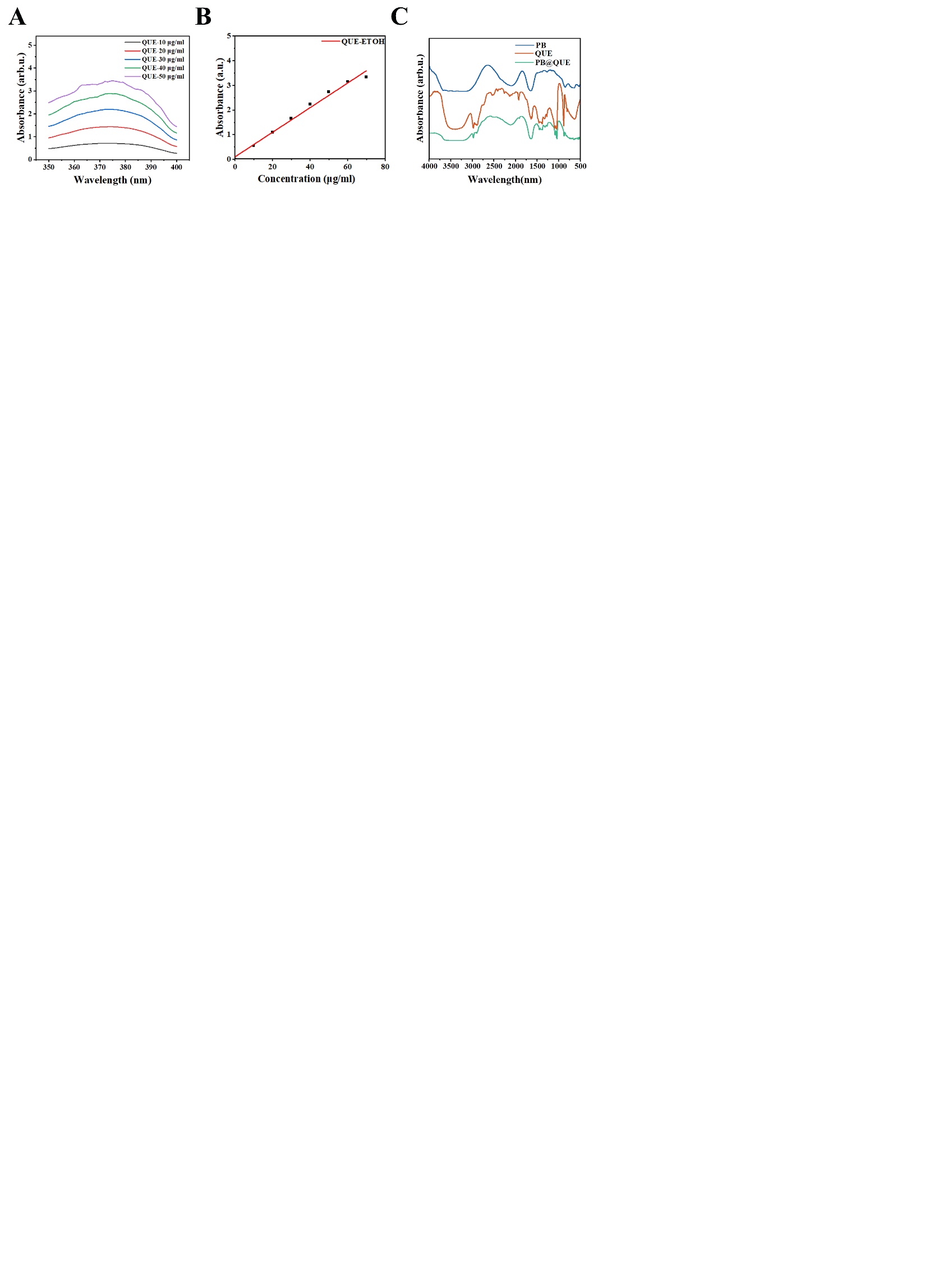


**Fig S1.** **The characterization of PB, QUE, and PB@QUE.** (A) UV absorbance spectra of QUE at different concentrations, (B) Concentration calibration curve based on the characteristic peak at 378 nm, (C) The infrared spectroscopy.


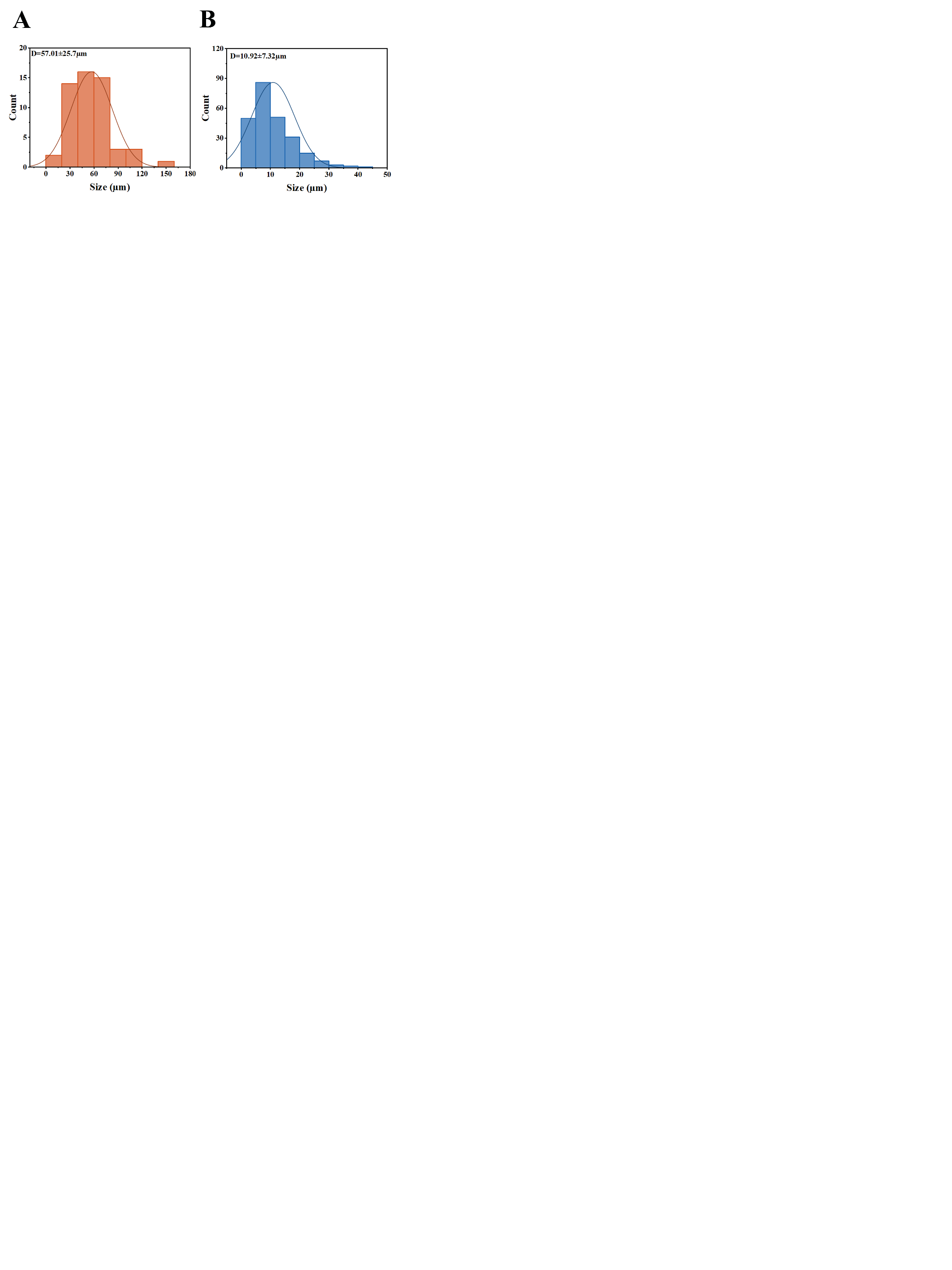


**Fig S2.** **The statistical analysis of pore diameters for (A) ATO and (B) ATO+PB samples.**


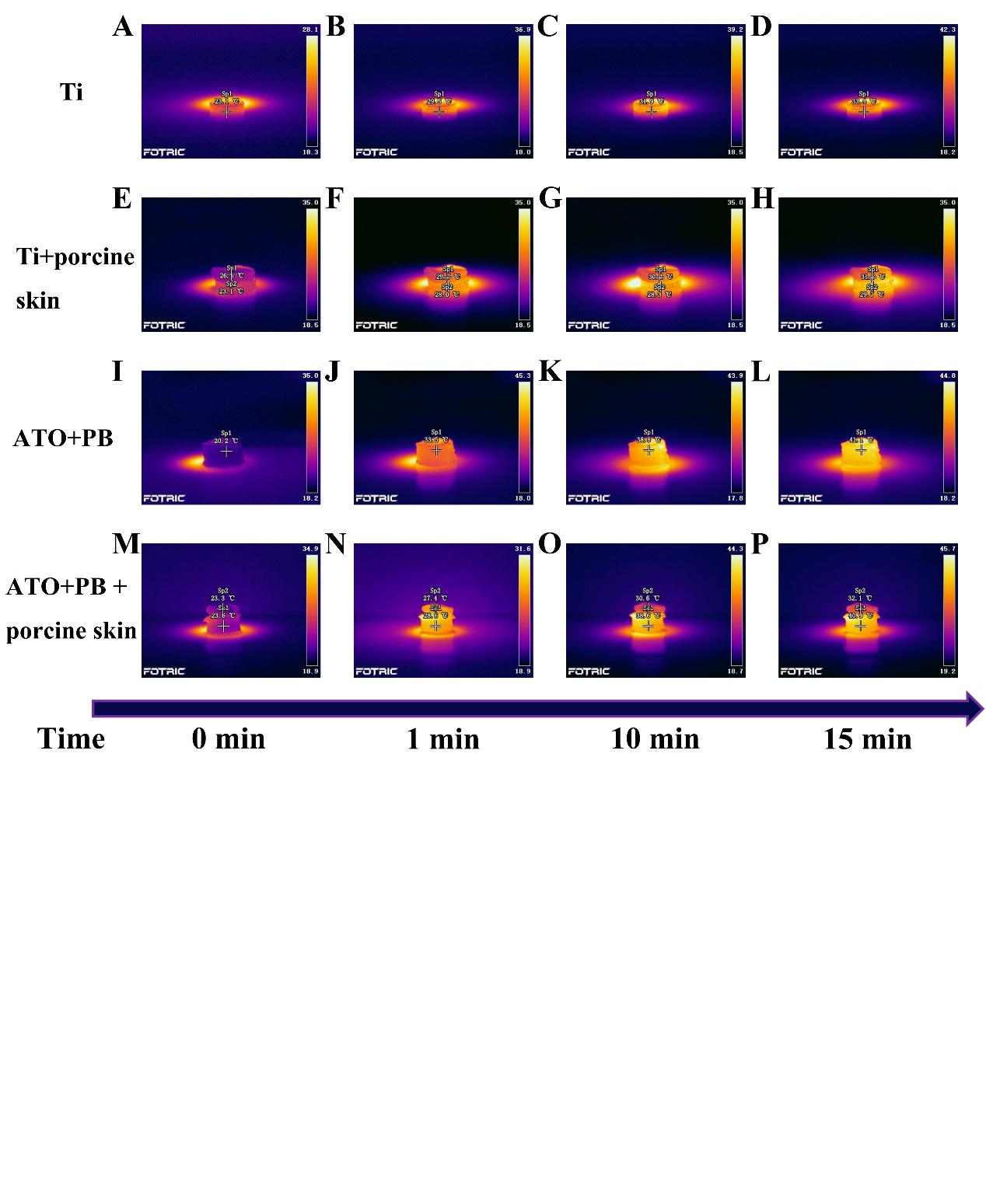


**Fig S3.** **Thermal images depicting the temperature variation of different samples over time under NIR irradiation**. (A)-(D): Ti; (E)-(H): Ti covered with porcine skin; (I)-(L): ATO+PB hydrogel; (M)-(P): ATO+PB hydrogel covered with porcine skin.


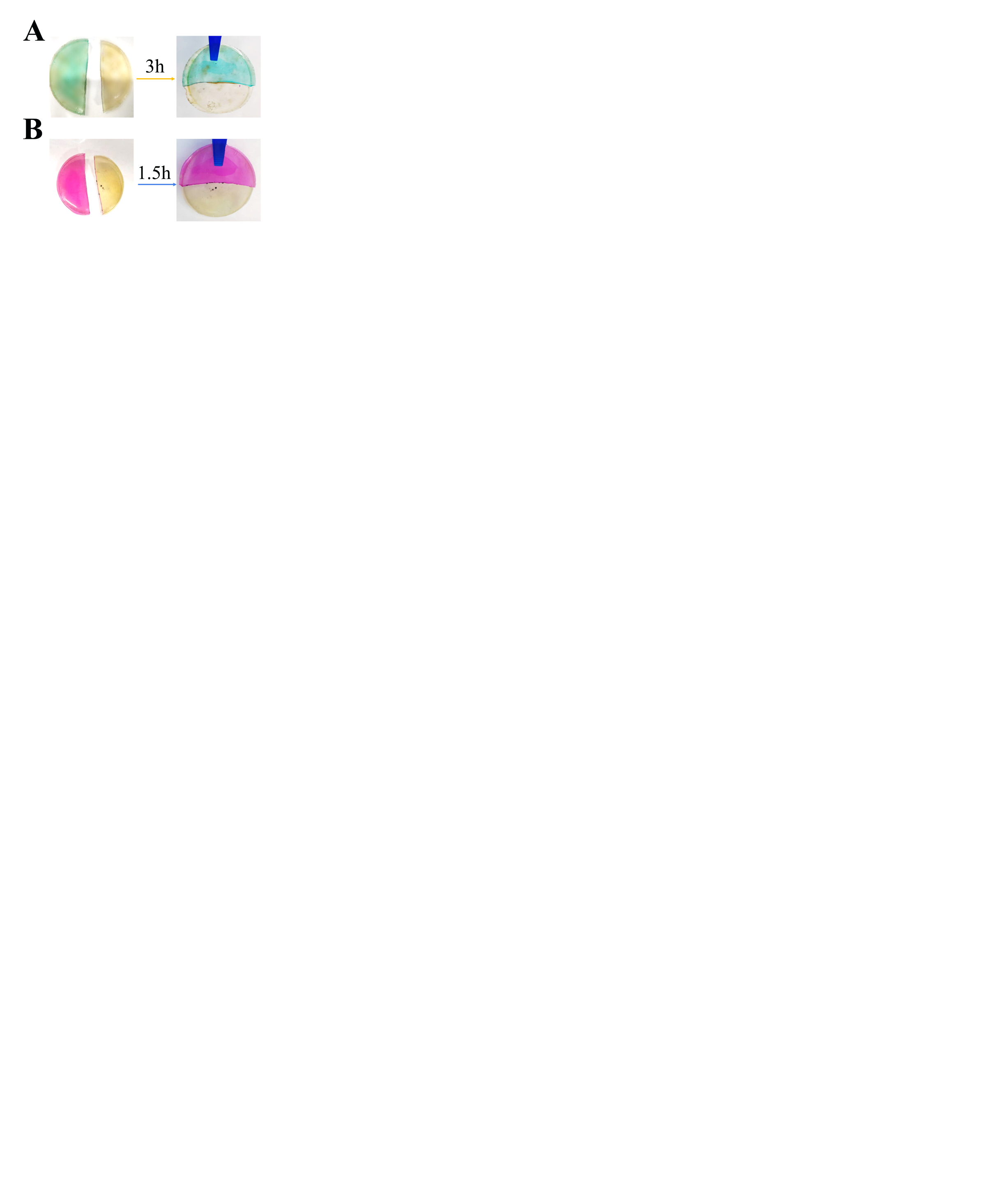


**Fig S4. Photographs of the self-healing of (A) ATO and (B) ATO+PB hydrogels at room temperature.**


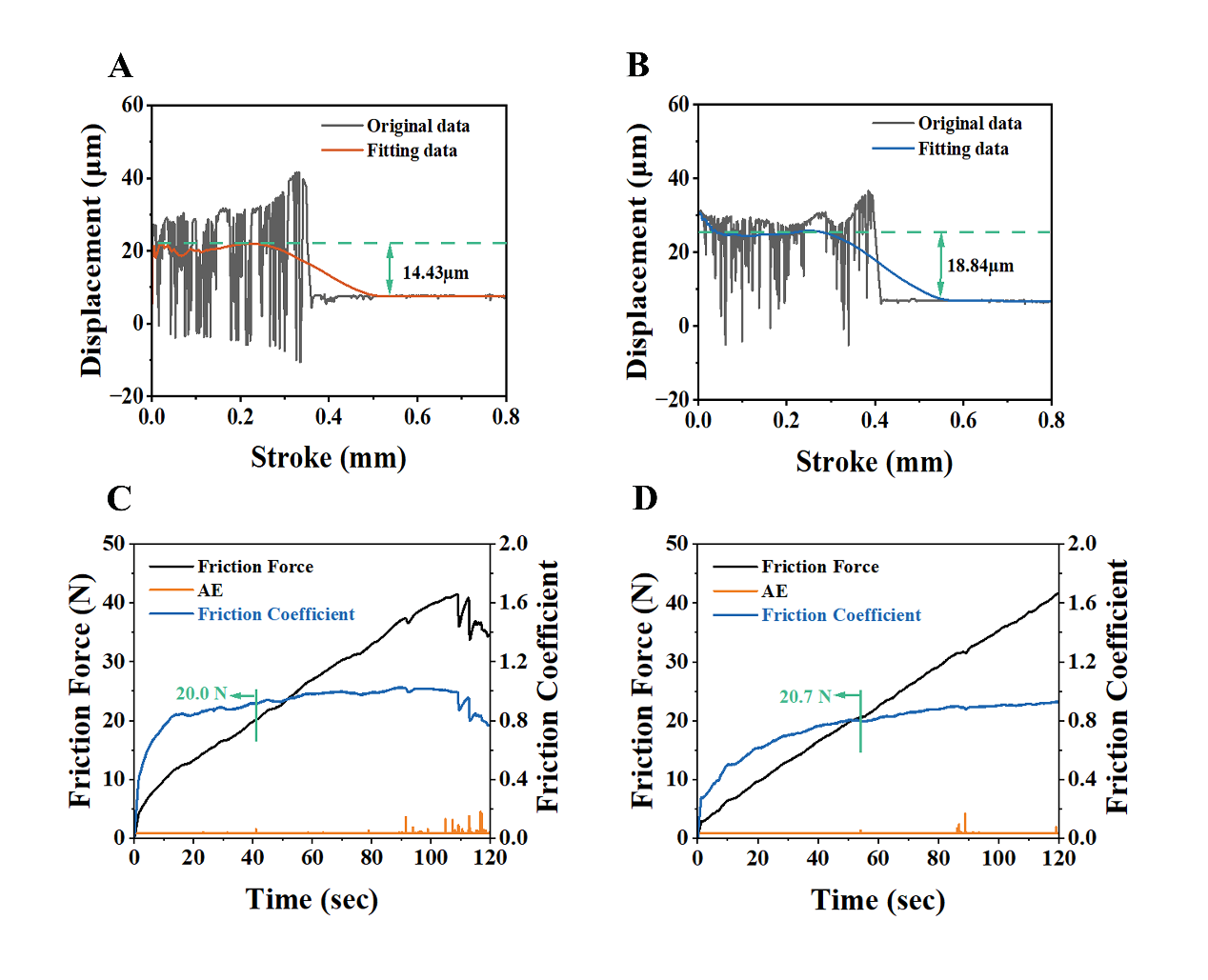


**Fig S5. Thickness and interfacial adhesion strength of different groups.** Thickness characterization of (A) ATO and (B) ATO+PB hydrogel coating deposited on Ti6Al4V substrate. Interfacial adhesion strength assessment between (C) ATO and (D) ATO+PB hydrogel coating and Ti6Al4V substrate.


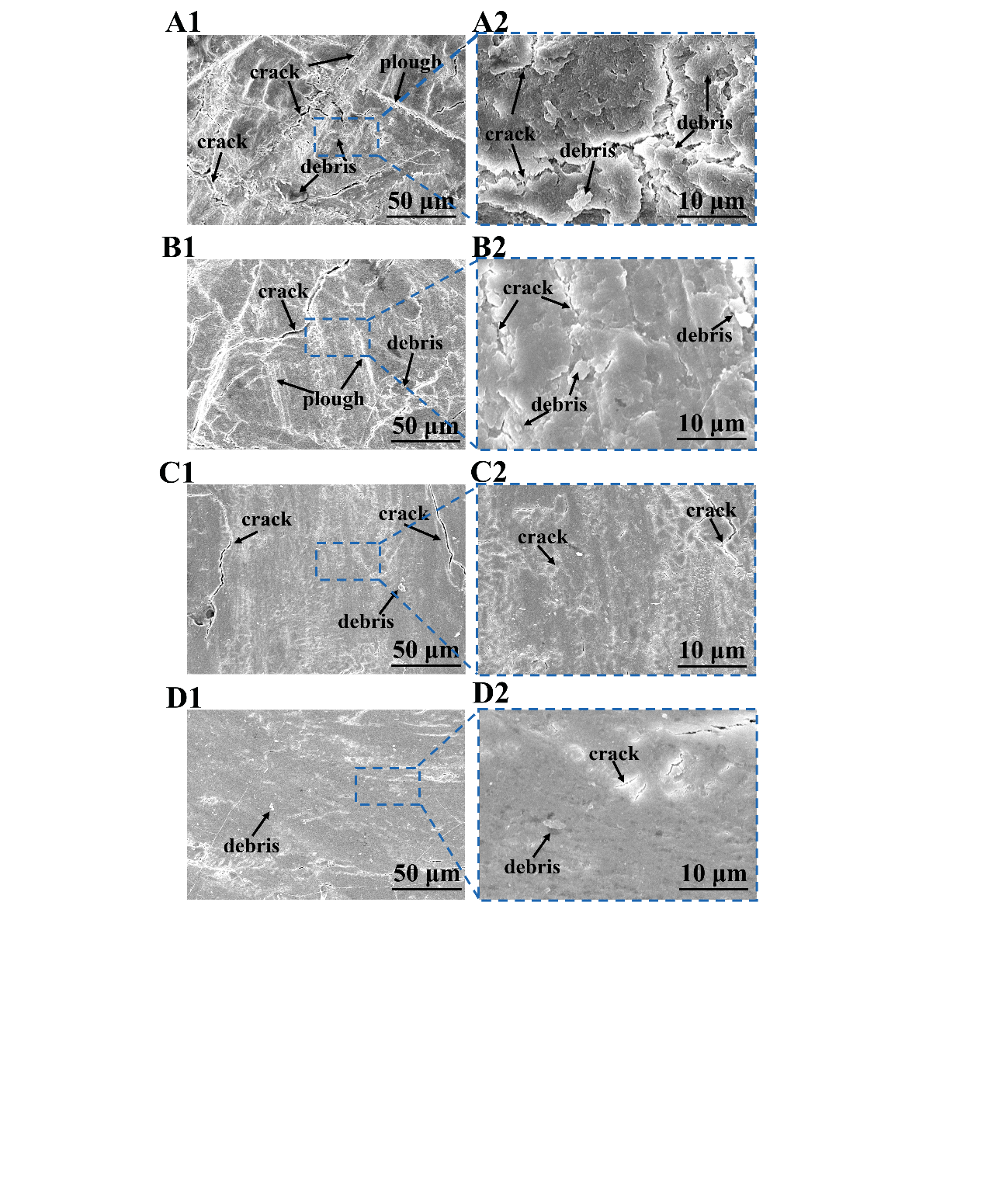


**Fig S6.** **SEM images of bone ball wear after friction tests against different samples (1: 500 ×, 2: 5000 ×).** A1-A2: Ti; B1-B2: ATO coating; C1-C2: ATO+PB coating; D1-D2: ATO+PB+NIR coating.


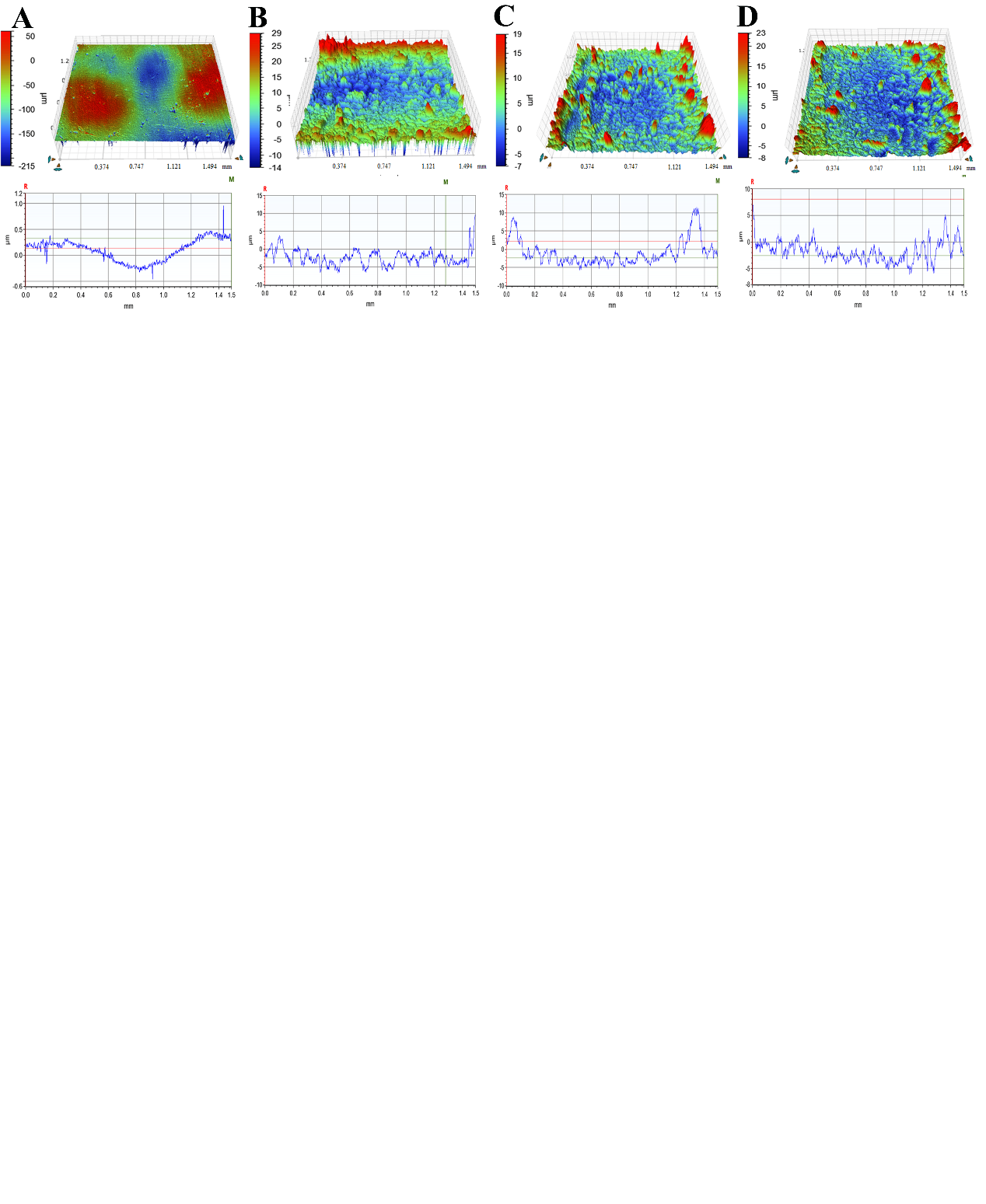


**Fig S7. Wear condition of the coating in the friction**. 3D white light interference images and corresponding wear depths of the lower friction pairs after friction. A: Ti; B: ATO coating; C: ATO+PB coating; D: ATO+PB+NIR coating.


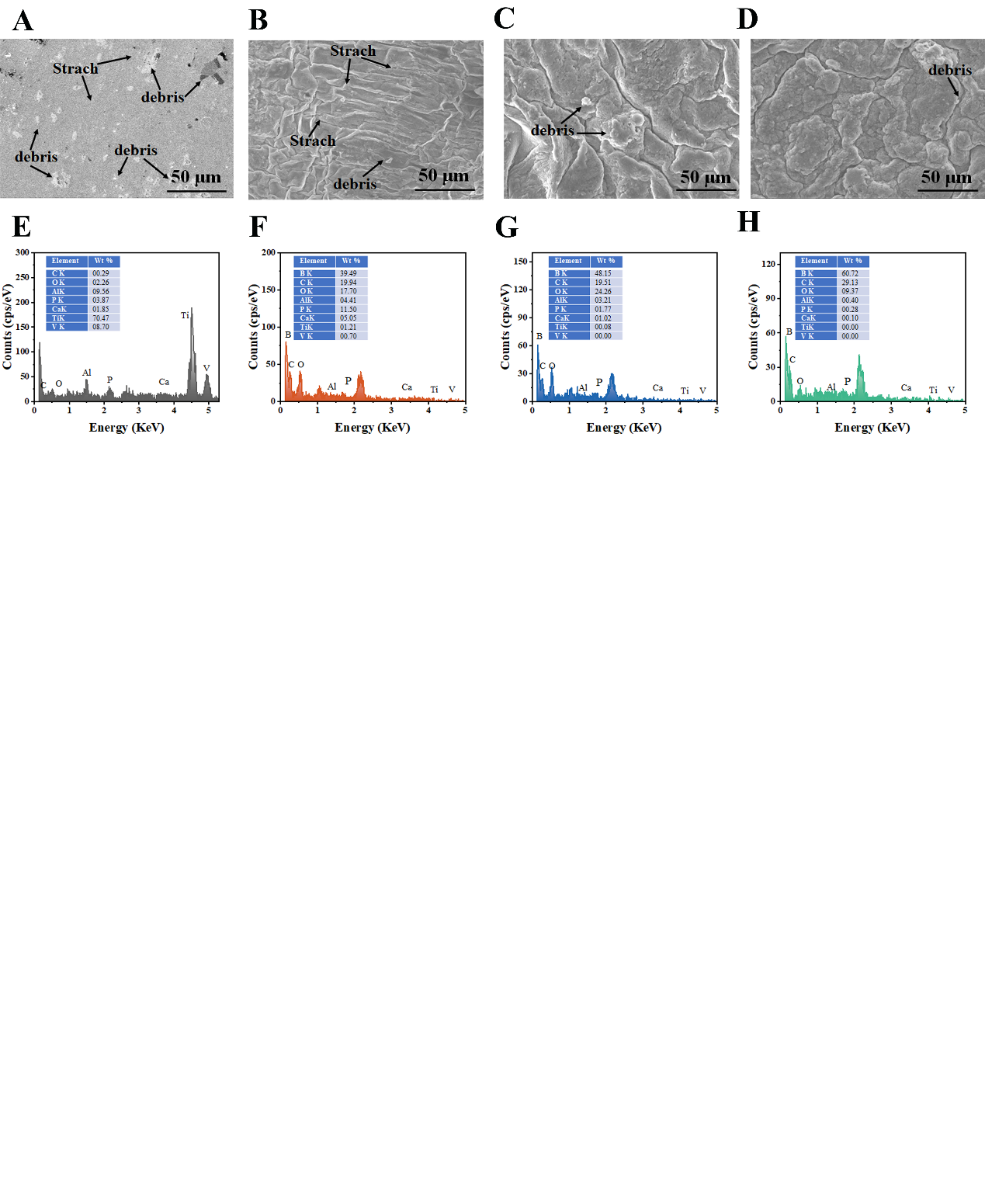


**Fig S8.** **SEM images (1000 ×) and corresponding EDS spectra of the lower friction pair after friction.** A and E: Ti; B and F: ATO coating; C and G: ATO+PB coating; D and H: ATO+PB+NIR coating.

**
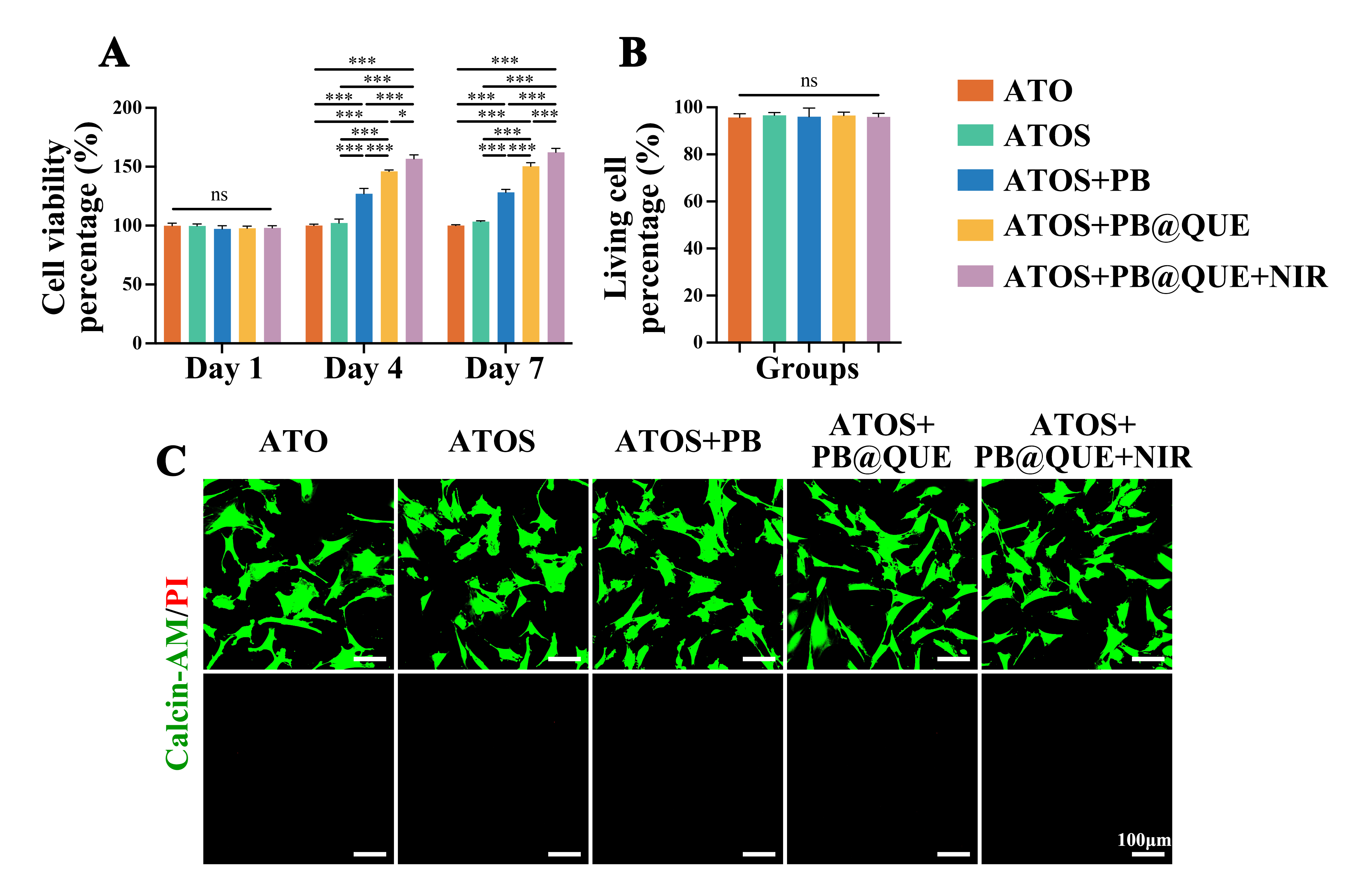
**

**Fig S9. Biocompatibility of the BMSCs after culturing with different groups’ extracts.** (A) Cell viability at day 1, 4 and 7 via CCK-8 assay. (B) Percentage of live cells at day 1 assessed by Live/Dead staining. (C) Representative fluorescence images of BMSCs obtained by Live/Dead staining. ^∗^ *p* < 0.05, and ^∗∗∗^ *p* < 0.001, n = 3.


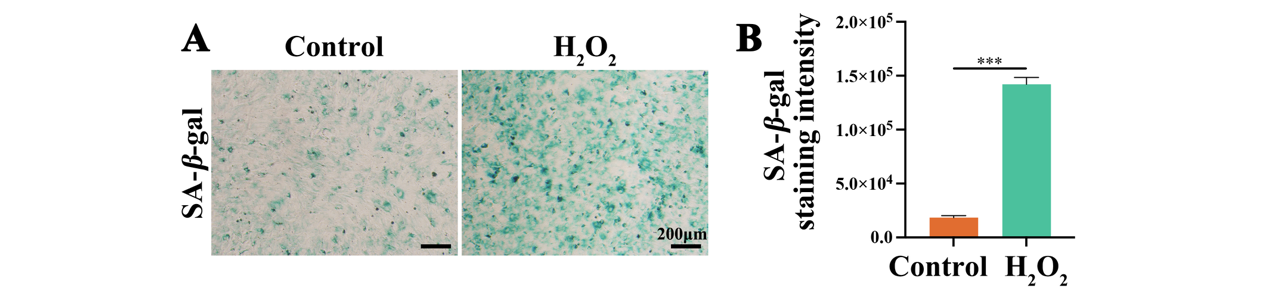


**Fig S10. The results of SA-*β*-gal staining of BMSCs.** (A) The representative images and (B) the statistical results of SA-*β*-gal staining of BMSCs in Control and H_2_O_2_ groups. ^∗∗∗^ *p* < 0.001, n = 3.


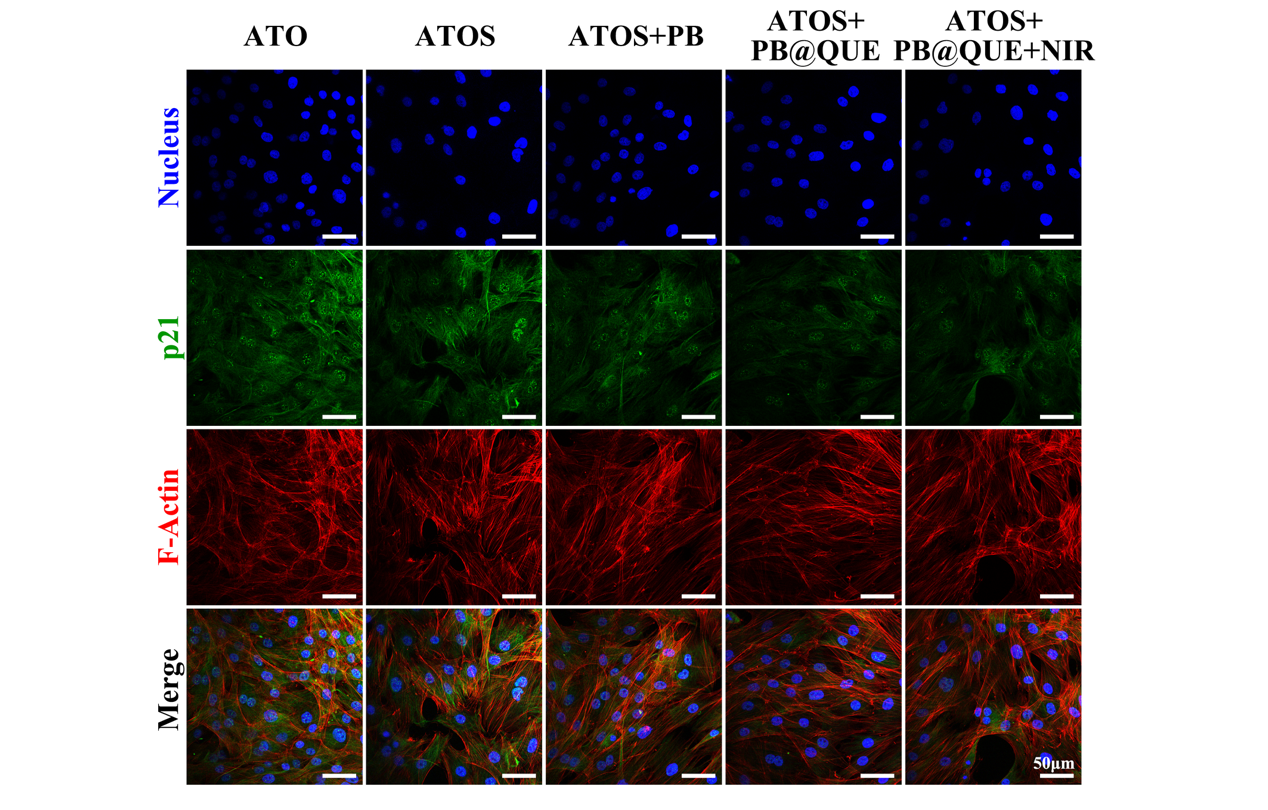


**Fig S11. The respresentative images of p21 staining in BMSCs in different groups.**


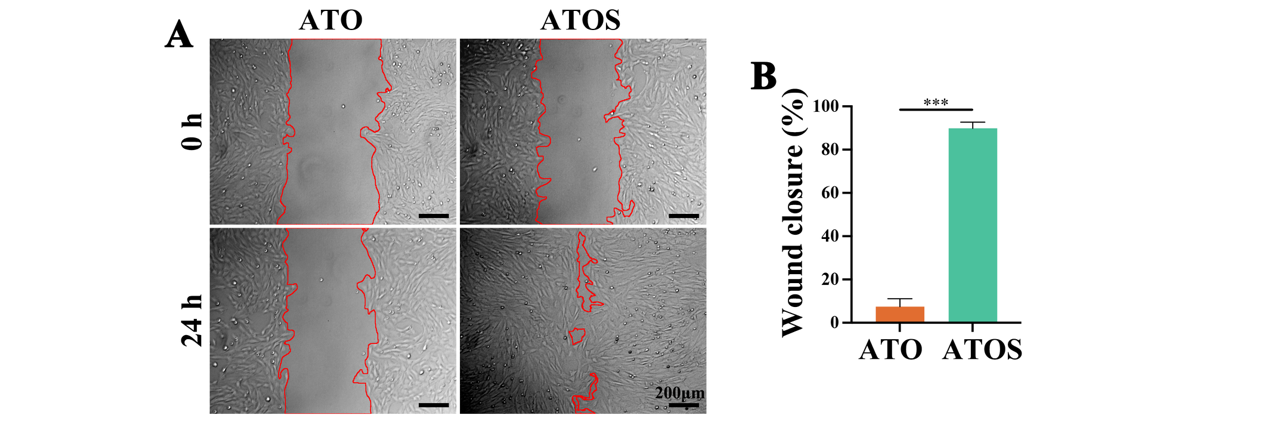


**Fig S12.** **The results of scratch assay of BMSCs.** (A) The representative images of scratch assay of BMSCs and (B) statistical results in ATO and ATOS groups. ^∗∗∗^ *p* < 0.001, n = 3.


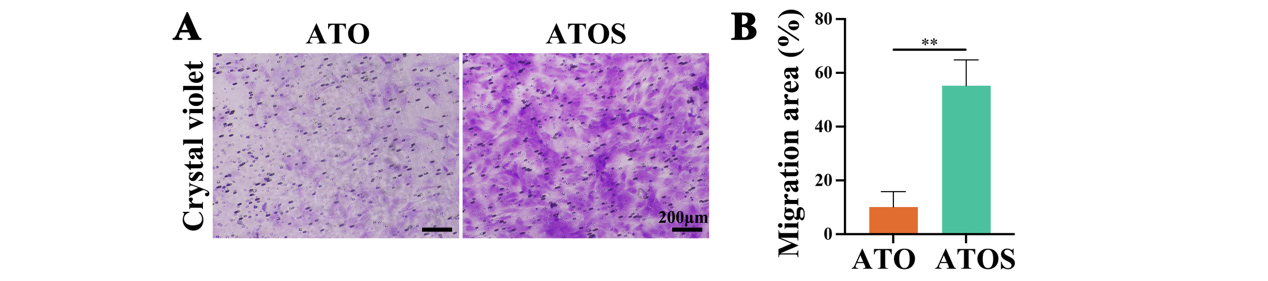


**Fig S13. The results of transwell assay of BMSCs.** (A) The representative images of transwell assay of BMSCs and (B) statistical results in ATO and ATOS groups. ^∗∗^ *p* < 0.01, n = 3.


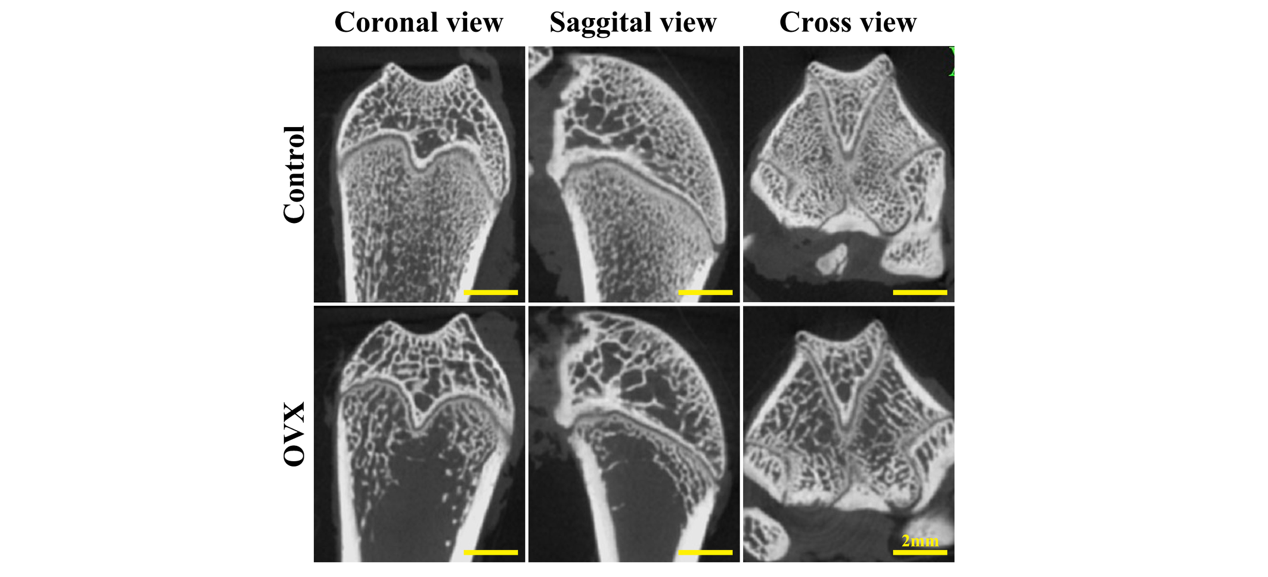


**Fig S14.** **The micro-CT images of femurs in rats in Control and OVX groups.**


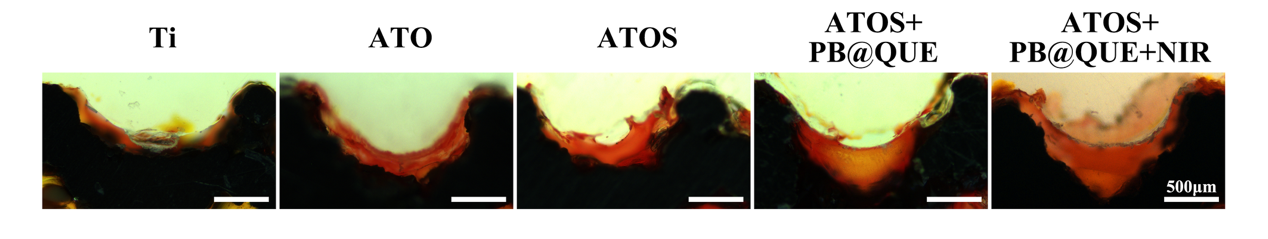


**Fig S15. VG staining of the collagen fiber at the bone-implant interface.**


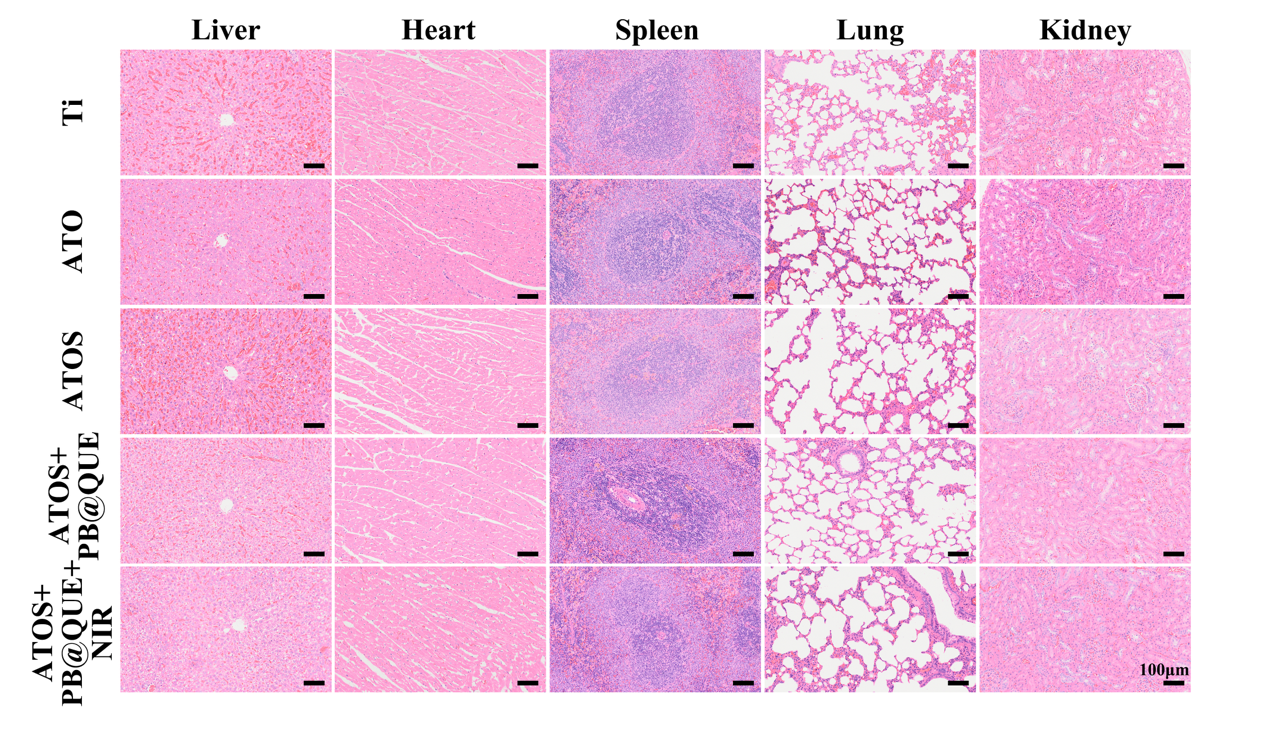


**Fig S16. The HE staining of livers, hearts, spleens, lungs and kidneys of rats in different groups.**

**Table S1** Composition of Ti Plate

| Composition | Al | V | Fe | Ti | C | H | N | O |
| --- | --- | --- | --- | --- | --- | --- | --- | --- |
| wt.% | 6.75 | 4.5 | 0.4 | 88.19 | 0.08 | 0.015 | 0.05 | 0.015 |

**Table S2** Concentrations of SBF solution

| Ion | SO_4_^2-^ | HPO_4_^2-^ | Mg^2+^ | Ca^2+^ | K^+^ | HCO_3_^-^ | Cl^-^ | Na^+^ |
| --- | --- | --- | --- | --- | --- | --- | --- | --- |
| Concentration (mM) | 0.5 | 1.0 | 1.5 | 2.5 | 5.0 | 10.0 | 103.0 | 122.5 |

**Table S3** Component category of the hydrogel coating.

| Sample | SDF-1α | PB | PB@QUE |
| --- | --- | --- | --- |
| ATO | - | - | - |
| ATO+PB | - | 40μg/mL | - |
| ATOS | 100 ng/mL | - | - |
| ATOS+PB | 100 ng/mL | 40μg/mL | - |
| ATOS+PB@QUE | 100 ng/mL | - | 40μg/mL |

**Table S4**. Sequences of primers for qRT-PCR.

| Gene | Primer |
| --- | --- |
| Raw264.7 (murine) | |
| *GAPDH-F* | AGGTCGGTGTGAACGGATTTG |
| *GAPDH-R* | TGTAGACCATGTAGTTGAGGTCA |
| *Il-4-F* | GGTCTCAACCCCCAGCTAGT |
| *Il-4-R* | GCCGATGATCTCTCTCAAGTGAT |
| *Il-10-F* | GCTCTTACTGACTGGCATGAG |
| *Il-10-R* | CGCAGCTCTAGGAGCATGTG |
| *iNOS-F* | GAGCCAGTCCTCTTTGCT |
| *iNOS-R* | CAACCTTGGTGTTGAAGGCG |
| *Il-6-F* | CTGCAAGAGACTTCCATCCAG |
| *Il-6-R* | AGTGGTATAGACAGGTCTGTTGG |
| BMSC (rat) | |
| *Gapdh-F* | TGTGTCCGTCGTGGATCTGA |
| *Gapdh -R* | TTGCTGTTGAAGTCGCAGGAG |
| *Opn-F* | AGCAAGAAACTCTTCCAAGCAA |
| *Opn-R* | GTGAGATTCGTCAGATTCATCCG |
| *Runx2-F* | GAGCGTTCAACGGCACAG |
| *Runx2-R* | GACAGTAGACTCCACGACA |
| *Col1-F* | AGAGGCATAAAGGGTCATCGTG . |
| *Col1-R* | AGACCGTTGAGTCCATCTTTGC |
| *Ocn-F* | GAGGACCCTCTCTCTGCTCA |
| *Ocn-R* | GGTAGCGCCGGAGTCTATTC |
| *p53-F* | GTTCGTGTTTGTGCCTGTCC |
| *p53-R* | TGCTCTCTTTGCACTCCCTG |
| *p16-F* | GTAGTACTGCACCAGGCAGG |
| *p16-R* | CCCAGCGGAGGAGAGTAGAT |
| *Tnf-α-F* | ATGGGCTCCCTCTCATCAGT |
| *Tnf-α-R* | GCTTGGTGGTTTGCTACGAC |
| *Il-1β-F* | GGCTGACAGACCCCAAAAGA |
| *Il-1β-R* | TGTCGAGATGCTGCTGTGAG |
| *Il-6-F* | CACTTCACAAGTCGGAGGCT |
| *Il-6-R* | TCTGACAGTGCATCATCGCT |
| *Ccl-2-F* | TGATCCCAATGAGTCGGCTG |
| *Ccl-2-R* | TGGACCCATTCCTTATTGGGG |
